# Effects of Immunization With the Soil-Derived Bacterium *Mycobacterium vaccae* on Stress Coping Behaviors and Cognitive Performance in a “Two Hit” Stressor Model

**DOI:** 10.3389/fphys.2020.524833

**Published:** 2021-01-05

**Authors:** Christine L. Foxx, Jared D. Heinze, Antonio González, Fernando Vargas, Michael V. Baratta, Ahmed I. Elsayed, Jessica R. Stewart, Kelsey M. Loupy, Mathew R. Arnold, M. C. Flux, Saydie A. Sago, Philip H. Siebler, Lauren N. Milton, Margaret W. Lieb, James E. Hassell, David G. Smith, Kyo A. K. Lee, Sandra A. Appiah, Evan J. Schaefer, Morgan Panitchpakdi, Nicole C. Sikora, Kelly C. Weldon, Christopher E. Stamper, Dominic Schmidt, David A. Duggan, Yosan M. Mengesha, Mikale Ogbaselassie, Kadi T. Nguyen, Chloe A. Gates, K’loni Schnabel, Linh Tran, Joslynn D. Jones, Martha H. Vitaterna, Fred W. Turek, Monika Fleshner, Pieter C. Dorrestein, Rob Knight, Kenneth P. Wright, Christopher A. Lowry

**Affiliations:** ^1^Department of Integrative Physiology, University of Colorado Boulder, Boulder, CO, United States; ^2^Center for Microbial Exploration, University of Colorado Boulder, Boulder, CO, United States; ^3^Department of Pediatrics, School of Medicine, University of California, San Diego, La Jolla, CA, United States; ^4^Center for Microbiome Innovation, University of California, San Diego, La Jolla, CA, United States; ^5^Collaborative Mass Spectrometry Innovation Center, Skaggs School of Pharmacy and Pharmaceutical Sciences, University of California, San Diego, La Jolla, CA, United States; ^6^Department of Psychology and Neuroscience, University of Colorado Boulder, Boulder, CO, United States; ^7^Center for Neuroscience, University of Colorado Boulder, Boulder, CO, United States; ^8^Center for Sleep and Circadian Biology, Department of Neurobiology, Northwestern University, Evanston, IL, United States; ^9^Department of Computer Science and Engineering, Jacobs School of Engineering, University of California, San Diego, La Jolla, CA, United States; ^10^Department of Bioengineering, Jacobs School of Engineering, University of California, San Diego, La Jolla, CA, United States; ^11^Veterans Health Administration, Rocky Mountain Mental Illness Research Education and Clinical Center, Rocky Mountain Regional Veterans Affairs Medical Center, Aurora, CO, United States; ^12^Military and Veteran Microbiome: Consortium for Research and Education, Aurora, CO, United States; ^13^Department of Physical Medicine and Rehabilitation and Center for Neuroscience, University of Colorado Anschutz Medical Campus, Aurora, CO, United States; ^14^inVIVO Planetary Health, Worldwide Universities Network, West New York, NJ, United States

**Keywords:** cognition, diurnal, locomotor activity, metabolome, microbiome, microbiome-gut-brain axis, microbiota, stress resilience

## Abstract

Previous studies demonstrate that *Mycobacterium vaccae* NCTC 11659 (*M. vaccae*), a soil-derived bacterium with anti-inflammatory and immunoregulatory properties, is a potentially useful countermeasure against negative outcomes to stressors. Here we used male C57BL/6NCrl mice to determine if repeated immunization with *M. vaccae* is an effective countermeasure in a “two hit” stress exposure model of chronic disruption of rhythms (CDR) followed by acute social defeat (SD). On day –28, mice received implants of biotelemetric recording devices to monitor 24-h rhythms of locomotor activity. Mice were subsequently treated with a heat-killed preparation of *M. vaccae* (0.1 mg, administered subcutaneously on days –21, –14, –7, and 27) or borate-buffered saline vehicle. Mice were then exposed to 8 consecutive weeks of either stable normal 12:12 h light:dark (LD) conditions or CDR, consisting of 12-h reversals of the LD cycle every 7 days (days 0–56). Finally, mice were exposed to either a 10-min SD or a home cage control condition on day 54. All mice were exposed to object location memory testing 24 h following SD. The gut microbiome and metabolome were assessed in fecal samples collected on days –1, 48, and 62 using 16S rRNA gene sequence and LC-MS/MS spectral data, respectively; the plasma metabolome was additionally measured on day 64. Among mice exposed to normal LD conditions, immunization with *M. vaccae* induced a shift toward a more proactive behavioral coping response to SD as measured by increases in scouting and avoiding an approaching male CD-1 aggressor, and decreases in submissive upright defensive postures. In the object location memory test, exposure to SD increased cognitive function in CDR mice previously immunized with *M. vaccae*. Immunization with *M. vaccae* stabilized the gut microbiome, attenuating CDR-induced reductions in alpha diversity and decreasing within-group measures of beta diversity. Immunization with *M. vaccae* also increased the relative abundance of 1-heptadecanoyl-sn-glycero-3-phosphocholine, a lysophospholipid, in plasma. Together, these data support the hypothesis that immunization with *M. vaccae* stabilizes the gut microbiome, induces a shift toward a more proactive response to stress exposure, and promotes stress resilience.

## Introduction

Stress-related psychiatric disorders, such as major depression, affect more than 17.3 million adults aged 18 and older in the United States each year (Substance Abuse, and Mental Health and Services Administration, 2018). One important risk factor for the development of stress-related psychiatric disorders, including major depressive disorder and anxiety disorders, is psychosocial stress (Fan et al., 2015). Despite evidence supporting the associations between psychosocial stress, depression, and anxiety, the biological mechanisms by which psychosocial stress and stress-related psychiatric disorders such as depression are related to one another are poorly understood. Studies suggest that chronic low-grade inflammation, particularly in response to lower subjective social status, may be an important factor with a causal role in the development of depression (Bellingrath et al., 2010; Rohleder, 2014; Eddy et al., 2016). In fact, multiple convergent lines of evidence in humans and in animal models suggest that exaggerated or inappropriate peripheral inflammation increases the risk of stress-related psychiatric disorders (Hodes et al., 2014; Miller and Raison, 2016). While a number of factors contribute to individual variability in peripheral proinflammatory immune responses, the microbiome has recently received considerable attention (Cryan and Dinan, 2012; Belkaid and Hand, 2014; Lowry et al., 2016; Flux and Lowry, 2019).

Throughout evolution, humans have coevolved with diverse microorganisms, including prokaryotes, eukaryotes, and viruses, which together constitute the human microbiome (Hooper et al., 2002; Dave et al., 2012). Specific microorganisms have been shown to prime immunoregulatory circuits and suppress pathological inflammation through the actions of regulatory T cells (Treg) (Rook et al., 2004). These include: (1) commensal microbiota that have been altered by the Western lifestyle (von Hertzen et al., 2015; Sonnenburg and Sonnenburg, 2019); (2) pathogens associated with “old infections” from the hunter-gatherer period of human evolution, including Paleolithic strains of *Mycobacterium tuberculosis* that were less pathogenic than extant ones (Comas et al., 2013), helminths including gut parasites (Babu et al., 2006), and *Helicobacter pylori* (Atherton and Blaser, 2009; reviewed in Rook, 2010; Rook et al., 2014); and (3) organisms from the natural environment, such as water- and soil-associated saprophytes including *Mycobacterium vaccae*, which were frequently encountered by humans and tolerated by the immune system (Rook et al., 2004). However, the human microbiome may have shifted radically as a result of living a modern urban lifestyle, with entire classes of these microorganisms that prime immunoregulatory circuits either reduced or absent (Rook et al., 2004; Blaser and Falkow, 2009; von Hertzen et al., 2011; Sonnenburg and Sonnenburg, 2019). Compositional alterations in the microbiome due to modern urban living are thought to alter the manner in which the peripheral immune system responds to challenge, resulting in a deficiency of Treg development, a shift toward imbalanced immunoregulation, chronic low-grade inflammation (Rook et al., 2004), and exaggerated proinflammatory responses to psychosocial stressors (Böbel et al., 2018).

Consistent with the “Old Friends” hypothesis, individuals raised in an urban environment have an increased proinflammatory immune response to psychosocial stress relative to individuals raised in a rural environment with farm animals (Böbel et al., 2018). Although many factors have been identified that may contribute to the industrialized microbiota (Sonnenburg and Sonnenburg, 2019), chronic low-grade inflammation (Rohleder, 2014), and vulnerability to exaggerated or prolonged inflammatory responses to psychosocial stress (Böbel et al., 2018), one common factor associated with the modern lifestyle is circadian disruption. Circadian disruption alters the microbiome (Voigt et al., 2014, 2016; Deaver et al., 2018; Wu et al., 2018; Parkar et al., 2019), induces chronic low-grade inflammation (Li et al., 2018; Inokawa et al., 2020), and induces exaggerated inflammatory responses to immune challenge (Castanon-Cervantes et al., 2010; Adams et al., 2013; Brager et al., 2013; Guerrero-Vargas et al., 2015; Comas et al., 2017). Circadian disruption, in turn, increases risk of stress-related psychiatric disorders (Karatsoreos, 2014). This reasoning has led to the development of strategies to promote stress resilience and to prevent or treat psychiatric disorders by restoring some of the lost “Old Friends” through microbial-based interventions. Repeated immunization with *Mycobacterium vaccae* NCTC 11659 (*M. vaccae*), one such microbial-based intervention, has been shown to shift immune signaling toward an immunoregulatory phenotype and prevent inappropriate inflammation (Lowry et al., 2016; Reber et al., 2016b; Fonken et al., 2018; Frank et al., 2018; Amoroso et al., 2019a,b; Loupy et al., 2019).

Here we evaluated the effects of immunization with *M. vaccae* NCTC 11659, a soil-derived bacterium with anti-inflammatory and immunoregulatory properties (Zuany-Amorim et al., 2002b), in a “two hit” model of stress exposure, incorporating stressors that are common in modern urban lifestyles such as circadian disruption and psychosocial stress. We evaluated the effects of *M. vaccae* immunization and chronic disruption of rhythms (CDR) on behavioral responses during acute social defeat (SD) that have been identified as determinants of individual variability in stress resilience and vulnerability to anxiety- and depressive-like behavioral responses (Koolhaas et al., 1999; Veenema et al., 2007; Wood et al., 2010; Wood and Bhatnagar, 2015). In addition, we assessed the effects of *M. vaccae*, CDR, and SD on subsequent cognitive performance in the object location memory (OLM) test. Finally, we evaluated potential mediators and moderators of the effects of *M. vaccae*, CDR, and SD, including measures of alpha and beta diversity in the gut microbiome, the fecal metabolome, the host plasma metabolome, and serotonergic gene expression in the midbrain and pontine raphe complex, brain systems implicated in individual variability in vulnerability to anxiety- and depressive-like behavioral responses (Wood et al., 2015; Hassell et al., 2018).

## Materials and Methods

### Animals and Housing

**FIGURE 1:**
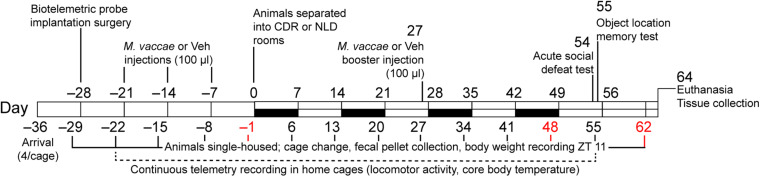
Diagrammatic illustration of the experimental protocol. White bars indicate normal 12-h light:dark cycling with lights on at 0600 MDT or 0700 h MST, black bars indicate light cycle reversal periods for the chronic disruption of rhythms (CDR) group. CDR, chronic disruption of rhythms; MDT, Mountain Daylight Time; MST, Mountain Standard Time; *M. vaccae, Mycobacterium vaccae* NCTC 11659; NLD, normal light:dark condition; Vehicle, borate-buffered saline vehicle; ZT, Zeitgeber time.

Seven consecutive cohorts of male C57BL/6NCrl mice (*N* = 112, Charles River Laboratories; six cohorts originated from the Raleigh, NC, United States facility and one cohort originated from the Kingston, NY, United States facility) arrived 28 days old on experimental day –36 (Figure 1). All mice experienced a phase delay of ∼2 h (Coordinated Universal Time (UTC)-05:00, Eastern Standard Time to UTC-07:00, Mountain Standard Time [MST]) and significant elevation gains during transport (1,559 m from Raleigh, NC, United States; 1,510 m from Kingston, NY, United States). Mice were group-housed upon arrival (4 mice/cage) in polycarbonate mouse cages with stainless steel wire bar lids (29.2 cm L × 18.4 cm W × 12.7 cm H, Alternative Design Manufacturing & Supply, Siloam Springs, AR, United States) and aspen chip bedding (Cat. no. 7093, Teklad Laboratory Grade Aspen Bedding, Envigo, Madison, WI, United States). Standard rodent chow (Cat. no. 2018, Teklad Global 18% Protein Rodent Diet, Envigo) and tap water were provided *ad libitum* for the duration of the experiment. Mice were entrained after arrival for 5 weeks to a standard 12-h light:dark (LD) cycle with lights on at 0600 h MST or 0700 h Mountain Daylight Time (MDT), then split evenly into one of two adjacent animal housing rooms for designated CDR or stable normal light:dark (NLD) conditions. Mice were separated into single-housing conditions on day –29 and otherwise kept under the same conditions as those described above for the remainder of the experiment. The research described here was conducted in compliance with The ARRIVE Guidelines for Reporting Animal Research (Kilkenny et al., 2010), and the *Guide for the Care and Use of Laboratory Animals*, Eighth Edition (Institute for Laboratory Animal Research, The National Academies Press, Washington, D.C., 2011), and was approved by the University of Colorado Boulder Institutional Animal Care and Use Committee and the Bureau of Medicine and Surgery. All efforts were made to minimize the number of animals used and their suffering.

### Experimental Design and Tissue Collection

The general experimental procedures for *M. vaccae* administration, CDR, and SD protocols are illustrated in Figure 1. All 112 mice in the study received surgical implantation of telemetric transmitters at Zeitgeber time (ZT) 4 on day –28 in order to continually record individual subjects’ locomotor activity (LA) levels throughout the experiment. Due to complications following surgery, 12 mice were removed from the study, resulting in *N* = 100 mice that were immunized with *M. vaccae* (*Mv*) or borate-buffered saline (BBS) vehicle (Veh) beginning on day –21. Data inclusion criteria used for analysis throughout 2015–2017 and calculated *n* values for each analysis are listed in Supplementary File S1. Cages were changed and fecal pellets were collected weekly on days –29, –22, –15, –8, and –1, prior to initiation of the CDR protocol. On days –21, –14, and –7, mice received subcutaneous injections of vehicle (100 μl, *n* = 47) or a whole-cell, heat-killed preparation of *Mv* (0.1 mg/ml in 100 μl BBS, *n* = 53) as in previous studies; an additional subcutaneous injection of *Mv* or Veh was given on day 27 because *Mv’*s effects on behavioral outcomes beyond 4 weeks have not been previously demonstrated.

On day 0, experimental mice given *Mv* or Veh were evenly split into rooms set to either NLD (*Mv*/NLD, *n* = 25; Veh/NLD, *n* = 23) or CDR conditions with weekly reversal of the 12-h LD cycle for 8 consecutive weeks (*Mv/CDR, n* = 24; Veh/CDR, *n* = 20). For each subsequent cohort of mice, the assignment of the two rooms to either NLD or CDR conditions was reversed in order to avoid systematic room effects. Mice in the CDR room experienced reversal of the LD cycle on a weekly basis beginning on day 0 (i.e., beginning on day 0, week 1, lights off at 0600 h MST or 0700 h MDT and lights on at 1800 MST or 1900 h MDT; beginning on day 7, week 2, lights on at 0600 MST or 0700 MDT and lights off at 1800 h MST or 1900 h MDT), and continuing through days 21, 28, 35, 42, 49, and 56 (Table 1). Mice in both NLD and CDR rooms were placed in fresh cages on days preceding weekly light reversals for fecal sample collections (days 6, 13, 20, 27, 34, 41, and 48).

**TABLE 1:** Details of the 8-week chronic disruption of rhythms protocol.

**Week**	**Days**	**24 h condition to initiate phase delay in CDR group**	**Lights on**	**Lights off**
1	0–6	24 h dark ending at 1800 h MDT or 1900 h MST on day 0	1800 h MDT or 1900 h MST	0600 h MDT or 0700 h MST
2	7–13	24 h light ending at 1800 h MDT or 1900 h MST on day 7	0600 h MDT or 0700 h MST	1800 h MDT or 1900 h MST
3	14–20	24 h dark ending at 1800 h MDT or 1900 h MST on day 14	1800 h MDT or 1900 h MST	0600 h MDT or 0700 h MST
4	21–27	24 h light ending at 1800 h MDT or 1900 h MST on day 21	0600 h MDT or 0700 h MST	1800 h MDT or 1900 h MST
5	28–34	24 h dark ending at 1800 h MDT or 1900 h MST on day 28	1800 h MDT or 1900 h MST	0600 h MDT or 0700 h MST
6	35–41	24 h light ending at 1800 h MDT or 1900 h MST on day 35	0600 h MDT or 0700 h MST	1800 h MDT or 1900 h MST
7	42–48	24 h dark ending at 1800 h MDT or 1900 h MST on day 42	1800 h MDT or 1900 h MST	0600 h MDT or 0700 h MST
8	49–55	24 h light ending at 1800 h MDT or 1900 h MST on day 49	0600 h MDT or 0700 h MST	1800 h MDT or 1900 h MST

*CDR, chronic disruption of rhythms; MDT, Mountain Daylight Time; MST, Mountain Standard Time.*

On day 54, a subset of mice selected from each treatment group underwent SD testing in a separate animal housing room with a screened CD-1 mouse aggressor for 10 min (Veh/NLD/SD, *n* = 13; Veh/CDR/SD, *n* = 8; *Mv*/NLD/SD, *n* = 11, *Mv*/CDR/SD, *n* = 11). Mice not assigned to the SD subset remained undisturbed in a single-housed home cage control condition (SHC) for the same period of time (Veh/NLD/SHC, *n* = 9; Veh/CDR/SHC, *n* = 10; *Mv/*NLD/SHC, *n* = 14; *Mv/*CDR/SHC, *n* = 9). Mice were placed in fresh cages immediately after the SD test on day 55 for an additional fecal sample collection. All mice were individually evaluated for hippocampal-dependent spatial memory in the OLM test 24 h following SD or SHC conditions for 5 min on day 55. Fecal samples from day –1 (BBS, *n* = 47; *M. vaccae*, *n* = 53), day 48 (Veh/NLD, *n* = 22; *Mv/*NLD, *n* = 18; Veh/CDR, *n* = 25; *Mv/*CDR, *n* = 20), and day 62 (Veh/NLD/SHC, *n* = 8; Veh/NLD/SD, *n* = 11; Veh/CDR/SHC, *n* = 8, Veh/CDR/SD, *n* = 5; *Mv/*NLD/SHC, *n* = 12; *Mv/*NLD/SD, *n* = 9; *Mv/*CDR/SHC, *n* = 7; *Mv/*CDR/SD, *n* = 9) were then shipped to the University of California, San Diego (UCSD) for 16S small subunit ribosomal RNA (16S rRNA) gene sequencing and liquid chromatography-mass spectrometry (LC-MS/MS) metabolomics analyses. Mice were euthanized by CO_2_ overdose on day 64 from ZT 2–4 followed by rapid decapitation to extract brains for *in situ* hybridization histochemistry analysis of serotonergic gene expression and to obtain aliquots of plasma for LC-MS/MS metabolomics analysis.

### Dates of Experimental Cohorts

Briefly, cohort 1 experimentation was conducted 12/14/2015–3/16/2016; cohort 2 experimentation was conducted 4/25/2016–8/3/2016; cohort 3 experimentation was conducted 8/29/2016–12/7/2016; cohort 4 experimentation was conducted 1/2/2017–4/12/2017; cohort 5 experimentation was conducted 7/3/2017–10/11/2017; cohort 6 experimentation was conducted 10/16/2017–1/24/2018; and cohort 7 experimentation was conducted 2/19/2018–5/30/2018.

### Continuous Monitoring of Environmental Conditions

One HOBO^®^ data-logging unit (Cat. no. U12-012, Onset Computer Corporation, Bourne, MA, United States) was placed in each animal housing room during the entire experiment to monitor temperature (in °C), relative humidity (as a %), and illuminance (in lux). At each location, thermocoupled sensors from the HOBO^®^ units were placed inside wall-mounted cases and were launched remotely by computer to sample ambient room temperature, relative humidity, and illuminance at 15-min intervals. Light levels were ∼185 lux when on, and ambient temperature and humidity were maintained in the ranges of 21–23°C and 14–18% respectively. Data from each HOBO^®^ unit was used to confirm valid LD cycles and to justify the removal of mouse samples from downstream statistical analyses due to adverse events such as spikes in room temperature because of ventilation failures or incorrect timing cycles of the LD cycles in both the CDR and NLD housing rooms. See Supplementary File S1 for additional details.

### Fecal and Environmental Sample Collection for Microbiome and Metabolome Analyses

In order to conduct longitudinal sampling and analysis of the fecal microbiome at each of the experimental time points above, 2–4 fecal pellets were collected from each mouse on a weekly basis during the entire duration of the experiment (Figure 1). Experimenters in full personal protective equipment (JDH, AE, PS, and DD) weighed mice and changed cages weekly at ZT 10–12 on days –29, –22, –15, –8, –1, 6, 13, 20, 27, 34, 41, 48, 55, 62, and 64 (Figure 1 and Supplementary Figure S1) to ensure fecal samples being collected were fresh and standardized to the specific time of collection. Fecal pellets were collected using sterile 26 ½ gauge needles (Cat. no. 305111, BD Biosciences, San Jose, CA, United States) following placement of each mouse in a clean cage with fresh bedding. One to two pellets were then placed in separate sterile 0.65 ml microcentrifuge tubes for microbiome and metabolomics analysis. Environmental samples (clean bedding, fresh rodent chow, and tap water) were also collected in separate sterile 0.65 ml microcentrifuge tubes for microbiome and metabolome analyses before mice were placed into clean cages. Tap water samples were collected directly from pre-filled sterile water bottles provided by the Office of Animal Research staff. All microbiome and metabolome samples were stored immediately at –80°C after collection until they were shipped to UCSD as indicated above for DNA extraction, 16S rRNA gene sequencing, and LC-MS/MS analysis. Samples from days –1, 48, and 62 were selected for shipment to UCSD to investigate the potential effects of *Mv* treatment (day –1), *Mv* treatment × CDR (day 48), and *Mv* treatment × CDR × SD (day 62) on microbiome and metabolome composition.

### Biotelemetric Probe Implantation

All mice received intraperitoneal implantations of biotelemetry devices on day –28 to allow continuous recording of LA. Sterile telemetric probes [PhysioTel^®^ Model no. TA-F10, Data Sciences International (DSI^TM^), Minneapolis, MN, United States] were implanted into the peritoneal cavity of mice under inhaled isoflurane anesthesia (5% initial, 2% maintenance in 100% oxygen). Briefly describing the surgery, the mouse was placed on its back and abdominal hair shaved following initial anesthesia. The skin was disinfected with 70% ethanol, and meloxicam (4 mg/kg, Boehringer-Ingelheim, Ridgefield, CT, United States) was administered subcutaneously immediately prior to surgery. A single 1-cm midline incision was made through the dermis and abdominal muscle wall, starting just caudal to the xiphoid process of the sternum. A telemetric probe was sterilized with Actril^®^ (Cat. no. 176-02-046, Minnetech, Minneapolis, MN, United States) and implanted into the peritoneal cavity. Following probe implantation, the abdominal wall was closed using 4-0 non-absorbable surgical silk in a simple 3-stitch interrupted pattern (Cat. no. SS-683G, Med-Vet International, Mettawa, IL, United States). The skin was sealed with Dermabond Advanced^®^ topical adhesive (2-octyl cyanoacrylate, Ethicon US LLC, Somerville, NJ, United States). The mouse was then placed on a 37°C heating pad until full recovery. After the surgery, all mice were single housed in clean cages with fresh bedding as described above.

### Biotelemetric Recording and Analytical Approach

Implanted probes were calibrated and matched to individual receivers (PhysioTel^®^ Receiver Model no. RPC-1, DSI) placed on each cage and chain-linked to matrices for each mouse. Locomotor activity was continuously monitored at a frequency of 128 Hz in 1-min intervals. Data recorded from the biotelemetry devices (Dataquest ART version 3.0, DSI^TM^) 6 days after surgery, from days –22 through day 54, were utilized for diurnal rhythm analysis (Supplementary Figure S2). Locomotor activity data were visualized as double-plotted actograms using ClockLab (ver. 6.0, Actimetrics, Wilmette, IL, United States) and the *rethomics* R package (Geissmann et al., 2019).

### Subcutaneous Treatment With *Mycobacterium vaccae* NCTC 11659

Experimental mice received repeated subcutaneous injections of either Veh or 0.1 mg of a whole-cell, heat-killed preparation of *Mv* (10 mg/ml suspension diluted to 1 mg/ml in sterile-filtered BBS, batch ENG 1, Bio Elpida, Lyon, France) with injection sites between the scapulae on days –21, –14, –7, and 27. Each immunization was performed using 21-gauge needles (Cat. no. 315165, BD Biosciences) between ZT 2–4 to ensure the transmission of larger particulates in the whole-cell suspension. Dilutions of *Mv* material from stock suspensions provided by Bio Elpida were prepared immediately prior to injections in a single siliconized 1.5 ml microcentrifuge tube (Cat. no. 416556, Bio Plas, San Rafael, CA, United States). The dose used (0.1 mg; estimated to be 1 × 10^8^ bacteria) was 1/10 of the dose used in human studies (1 mg) (O’Brien et al., 2004), and was identical to the dose used in previous studies in mice and rats (Lowry et al., 2007; Reber et al., 2016b; Bowers et al., 2017; Fox et al., 2017; Fonken et al., 2018; Siebler et al., 2018; Amoroso et al., 2019a,b; Bowers et al., 2019; Hassell et al., 2019a; Loupy et al., 2019).

### Acute Social Defeat Testing

Retired breeder CD-1 male mice (∼17 weeks old or older, *n* = 70, Charles River Laboratories, Wilmington, MA, United States) were screened over three consecutive days from ZT 2–6 as aggressors for the SD paradigm. In each screening session, C57BL/6NCrl mice matched in age to mice in the study were placed directly into the home cage of each single-housed CD-1 mouse for 10 min. Latency to initial attack was recorded in s; if no attack took place during the screening session, this information was also recorded and used for CD-1 aggressor exclusion. For each of the following screening sessions, each CD-1 mouse was exposed to a novel screener C57BL/6NCrl mouse and latency to initial attack < 180 s was recorded. For each cohort of 16 mice, up to 5 CD-1 mice were selected as aggressors for SD testing in the study based on: attack behaviors displayed in at least 2 consecutive sessions, latency to initial attack < 60 s, and no incidence of physical injury or open wounds in screeners caused by potential aggressors.

CD-1 aggressors were selected for SD testing at least 1 week prior to day 54 in the experimental timeline. On day 54, single-housed C57BL/6NCrl mice in the study designated for SD testing as described above underwent acute SD testing in a separate animal housing room containing up to 4 CD-1 mouse aggressors. In the test, similar to the screening sessions, a single C57BL/6NCrl mouse was placed directly into the home cage of a single-housed CD-1 aggressor for 10 min (Veh/NLD/SD, *n* = 13; Veh/CDR/SD, *n* = 8; *Mv/*NLD/SD, *n* = 11, *Mv/*CDR/SD, *n* = 11). After 10 min, the C57BL/6NCrl mouse was removed and placed back in its home cage. C57BL/6NCrl mice not in the SD subset were subjected to a SHC control condition in which mice remained in their home cages within the NLD or CDR housing rooms throughout the SD testing period (Veh/NLD/SHC, *n* = 9; Veh/CDR/SHC, *n* = 10; *Mv/*NLD/SHC, *n* = 14; *Mv/*CDR/SHC, *n* = 9).

Behaviors were recorded in .mpg file format using a tripod-mounted digital video recorder (Carl Zeiss 1,8/1,8-108 Vario-Tessar lens, Model no. DCR-SX45, Sony Corp., Beijing, China) placed in front of the cages and later scored using a behavioral analysis computer software package (The Observer^®^ XT, Noldus Ltd., Wageningen, The Netherlands). Behaviors displayed by the C57BL/6NCrl mice were scored individually as described in Table 2 (adapted from Foertsch et al., 2017); the absence of any behavior listed above was scored as inactivity. All behaviors were scored individually by two experimenters blinded to treatment groups (AE and JS) for the duration of scoring with an inter-rater reliability of 0.975 or higher for each behavior described above.

**TABLE 2:** Behavior patterns scored during acute social defeat exposure.

**Behavior**	**Definition**
Attacking	A lunge at the CD-1 dominant male mouse with physical contact occurring
Avoiding; actively maintaining distance from the CD-1 dominant male	Directed movement away from the CD-1 dominant male mouse at walking speed, occurring before direct contact with the respective individual
Chasing	Following the fleeing CD-1 dominant male mouse at running pace
Mounting	Standing with two forepaws on the back of the CD-1 dominant male mouse, frequently resembling male-female copulation
Scouting	An attempt (stretched-attend posture or exploring at walking speed) to determine whether the threat (the CD-1 dominant male mouse) is still present
Flight	Directed movement away from the CD-1 dominant male mouse at running pace
Submissive upright posture	Standing on the hind legs, raising the forelimbs and presenting the belly to the threat (the CD-1 dominant male mouse)
Attacks received	Being jumped at by the CD-1 dominant male mouse with physical contact occurring
Chasing received	Being followed by the CD-1 dominant male mouse at running pace
Mounts received	Receiving the two forepaws of the CD-1 dominant male mouse on the back
Inactivity	The absence of any other behavior listed above

### Hippocampal-Dependent Spatial Memory Testing

The OLM test is a spatial learning and memory test without reinforcing cues shaping the acquisition process (Murai et al., 2007). After mice were acclimated to the behavioral testing room in their home cages for 1 h, an individual mouse was placed in the center of an open-field arena (90 lux in center, 45 cm width × 45 cm length × 40 cm height; Cat. no. LE802S, Bioseb, Chaville, France) containing two solid black phenolic laboratory bottle cap objects in adjacent corners, 2.5 cm away from the walls of the arena. The mouse was allowed to freely explore the arena for 5 min during the pre-test phase (Acquisition) before being returned to its home cage, whereupon the arena was cleaned with 70% ethanol and paper towels. Ninety min after the initial exposure, one of the two objects was displaced, such that the two objects were placed in diagonal corners, and the mouse was placed back in the arena for 5 min as before for the test phase (Retrieval). Objects moved (A1 or A2) were counterbalanced between mice/testing sessions.

Behaviors were recorded in .mpg file format using a tripod-mounted digital video recorder (Carl Zeiss 1,8/1,8-108 Vario-Tessar lens, Model no. DCR-SX45, Sony Corp.). From each video, time (s) spent exploring each object as defined by nose-point orientation toward the object within a 2.5 cm contact zone during each phase of the OLM test, relative to total time spent in the arena, was analyzed using a behavioral analysis computer software package (EthoVision^TM^ XT version 14, Noldus Ltd.). The data were then used to calculate time spent exploring objects during acquisition and retrieval, and the location index was calculated as the percentage of time spent exploring the displaced object relative to the time spent exploring both objects during retrieval for each individual mouse in the OLM test.

### Bacterial DNA Extraction and Generation of 16S rRNA Gene V4 Amplicons

Bacterial genomic DNA extraction, hypervariable region 4 (V4) amplicon generation from the 16S rRNA gene, and amplicon preparation for sequences were performed according to protocols benchmarked for the Earth Microbiome Project^1^. Briefly, bacterial genomic DNA was extracted from samples using the PowerMag DNA isolation kit optimized for KingFisher Duo^®^ (Cat. No. 27200-4, Mo Bio Laboratories, Carlsbad, CA, United States) according to manufacturer’s instructions. Marker genes in isolated DNA were polymerase chain reaction (PCR)-amplified in triplicate from each sample, targeting V4 of the 16S rRNA gene, modified with a unique 12-bp sequence identifier for each sample and the Illumina adapter, as previously described by Caporaso et al. (2012).

The PCR mixtures contained 13 μl Mo Bio PCR water, 10 μl 5′-HotMasterMix, 0.5 μl each of the barcoded forward and reverse primers (515-bp forward: 5′-GTGCCAGCMGCCGCGGTAA-3′; 806-bp reverse: 5′-GGACTACHVGGGTWTCTAAT-3′; Caporaso et al., 2012; 10 μM final concentration, Integrated DNA Technologies, San Diego, CA, United States), and 1.0 μl genomic DNA. Reaction mixtures were held at 94°C for 3 min, followed by 35 cycles of amplification (94°C for 45 s, 50°C for 1 min, and 72°C for 1.5 min), followed by a final extension at 72°C for 10 min. After amplification, the DNA concentration was quantified using PicoGreen^TM^ double-stranded DNA (dsDNA) reagent in 10 mM Tris buffer (pH 8.0, Cat. no. P11496, Thermo Fisher Scientific). A composite sample for sequencing (16S rRNA gene library) was created by combining equimolar ratios of amplicons from the individual samples, followed by ethanol precipitation to remove any remaining contaminants and PCR artifacts.

### 16S rRNA Gene Sequence and Data Preparation

Pooled amplicons were sequenced at the Institute for Genomic Medicine at UCSD using the Illumina MiSeq^®^ platform. The 16S rRNA gene library concentration was measured using the HiSens Qubit dsDNA HS assay kit (Cat. No. Q32854, ThermoFisher Scientific, Waltham, MA, United States). A total of 6 pM of the 16S rRNA gene library combined with 0.9 pM (15%) PhiX sequencing library control v3 (Cat. no. FC-110,3001, Illumina Inc., San Diego, CA, United States) was sequenced with 150-bp paired-end reads on an Illumina MiSeq^®^ sequencing system using a MiSeq reagent kit v2 (300 cycles; Cat. no. MS-102-2002, Illumina Inc.). FASTQ files for reads 1 (forward), 2 (reverse), and the index (barcode) read were generated using the BCL-to-FASTQ file converter bcl2fastq (ver. 2.17.1.14, Illumina, Inc.).

Sequencing data were prepared and analyzed using the Quantitative Insights Into Microbial Ecology microbiome analysis pipeline (QIIME2 ver. 2020.2, Bolyen et al., 2019)^2^. Mapping files and raw sequencing information are publicly available on the microbiome study management platform Qiita^3^ (Gonzalez et al., 2018) and EMBL-EBI (Accession no. ERP015380). Raw sequencing results were quality-filtered and de-multiplexed using the Deblur algorithm (Amir et al., 2017) default parameters as follows: no ambiguous bases allowed, only one barcode mismatch allowed, and a minimum required Phred quality score of 3. Quality filtering resulted in an output feature table of 2,250,000 high-quality reads in 150 samples at 15,000 sequences per sample following rarefaction.

### Fecal Pellet Extraction for Metabolomics Analysis

The metabolomics data for this project are publicly available under MassIVE dataset IDs MSV000082650 for the fecal metabolomics dataset and MSV000082649 for the plasma metabolomics dataset on the Global Natural Products Social Molecular Networking (GNPS^4^; Wang et al., 2016). MS data management platform. Fecal pellets were extracted as described previously (Thompson et al., 2020). Each fecal pellet was weighed and transferred to 2.0 ml round-bottom microcentrifuge tubes (Cat. no. 990381, Qiagen, Germantown, MD, United States), to which a clean stainless-steel bead (Cat. no. 69989, Qiagen) and 1.5 ml of chilled extraction solvent (50% methanol in water, LC-MS grade; Cat. nos. A4564 and W64, respectively; ThermoFisher Scientific) were added. Samples were homogenized for 5 min at 25 Hz using a TissueLyser II system (Cat. no. 85300, Qiagen) and allowed to incubate for 20 min at –20°C before centrifugation at 14,000 rpm for 15 min at 4°C. A 1.2 ml aliquot of the resultant methanol extract was transferred to a Nunc 2.0 ml DeepWell plate (Cat. no. 278743, ThermoFisher Scientific) and frozen at –80°C prior to lyophilization using a centrifugal evaporator (FreeZone 4.5 L Benchtop Freeze Dryer with Centrivap Concentrator, Labconco, Kansas City, MO, United States). Extracts were redissolved in 200 μl of resuspension solvent (50% methanol + 2.0 μM sulfadimethoxine, CAS no. 122-11-2, Sigma-Aldrich, St. Louis, MO, United States), vortexed for 30 s and centrifuged at 2000 rpm for 15 min at 4°C. Samples (150 μl) were transferred to a new 96-well plate (Cat. no. 353263, Corning, Corning, NY, United States) and stored at 4°C for up to 24 h before LC-MS/MS analysis. A resuspension solvent QC and a six standard mix QC (50% MeOH spiked with 1.0 μM sulfamethazine, 1.0 μM sulfamethizole, 1.0 μM sulfachloropyridazine, 1.0 μM amitryptyline, and 1.0 μM coumarin 314) was run every 12th sample to assess sample background, carry over, chromatography behavior, peak picking and plate effects.

### Plasma Extraction for Metabolomics Analysis

Plasma samples were extracted by adding 100% methanol spiked with 2 μM sulfamethazine to a final concentration of 80%. The samples were vortexed for 2 min and then placed in –20°C for 20 min to aid in protein precipitation. Samples were then centrifuged at 15,000 rpm and 80% of the solvent volume was placed into a Nunc 2.0 ml DeepWell plate (Cat. no. 278743, ThermoFisher Scientific) and frozen at –80°C prior to lyophilization. Lyophilization was conducted as described above for fecal pellet extraction. For analysis, the dried samples were resuspended in 50% methanol spiked with 1 μM sulfadimethoxine.

### Mass Spectrometry Analysis of Fecal and Plasma Extracts

Aliquots of 5.0 μl of fecal extracts (one per ionization mode) or plasma were injected into a Vanquish ultra-high performance liquid chromatography system coupled to a Q-Exactive hybrid quadrupole-Orbitrap mass spectrometer (ThermoFisher Scientific, Bremen, Germany) fitted with a heated electrospray ionization (HESI-II) probe source controlled by Thermo SII for Xcalibur software (ThermoFisher Scientific). Reverse-phase chromatographic separation was achieved using a C18 core-shell column (Kinetex, 50 × 2.1 mm, 1.7 μm particle size, 100 Å pore size, Phenomenex, Torrance, CA, United States) held at 40°C with a flow rate of 0.5 ml/min (solvent A: H_2_O + 0.1% formic acid, solvent B: acetonitrile + 0.1% formic acid). 5.0 μL aliquots were injected per sample/QC. After injection, the samples were eluted using a linear gradient set as follows: 0–1 min, 5% B; 1–8 min, ramp from 5 to 100% B; 8–10 min, holdout phase at 100% B; 10–10.5 min, returned to 5% B; 10.5–12.5 min, re-equilibration phase at 5% B.

An external calibration with Pierce^TM^ LTQ Velos electrospray ionization (ESI) positive ion calibration solution (Cat. no. 88323, ThermoFisher Scientific) was performed prior to data acquisition with an error rate of less than 1 ppm. ESI positive ion mode was used to convert solution-phase molecules into gas-phase ions for MS analysis using the following source parameters: drying gas, 9.0 L/min; dry gas heater, 200°C; capillary voltage, +4.5 kV; end plate offset, –0.5 kV; and nebulizer, 2.0 bar. Positive electrospray ionization probe settings were as follows: sheath gas (N_2_) pressure of 52 lb/in^2^, auxiliary gas (N_2_) pressure of 14 lb/in^2^, sweep gas (N_2_) pressure of 3 lb/in^2^, spray voltage of 3.5 kV, capillary temperature of 270°C, S-lens radio frequency (RF) level of 50 Hz, and auxiliary gas heater temperature of 435°C. Negative electrospray ionization probe settings were as follows: sheath gas (N_2_) pressure of 52 lb/in^2^, auxiliary gas (N_2_) pressure of 14 lb/in^2^, sweep gas (N_2_) pressure of 3 lb/in^2^, spray voltage of 3.5 kV, capillary temperature of 270°C, S-lens RF level of 50 Hz, and auxiliary gas heater temperature of 435°C.

### LC-MS/MS Data Conversion and Upload Parameters

Product ion spectra were recorded in data-dependent acquisition (DDA) mode from 0.5 to 12 min. Fecal sample IDs were manually uploaded into an electronic spreadsheet and subsequently used to assign file names during LC-MS/MS data acquisition of product ion spectra. A full scan at MS^1^ level was performed with resolution (R) of 35,000 *m/z* in profile mode. We acquired MS/MS spectra in top 5 DDA mode, in which up to 5 of the most abundant ions of MS^1^ spectra obtained via survey scan (80–1200 *m/z* at 3 Hz) were subjected to normalized collision-induced dissociation (CID) at 30 eV. MS^2^ fragmentation scans produced by CID were performed at 17,500 resolution with a maximum ion injection time of 60 ms in profile mode, resulting in iterative scan cycles and several thousand spectra per sample. To reduce redundancy and computation time we clustered identical spectra to consensus spectra (Frank et al., 2008) and searched them against several spectral libraries, including GNPS (Wang et al., 2016), MassBank (Horai et al., 2010), ReSpect (Sawada et al., 2012), Human Metabolomics Database (HMDB; Forsythe and Wishart, 2009), and National Institutes of Standards and Technology (NIST; Stein, 2014). Raw data files were converted to the .mzXML file format using MZMine version 2.3^5^ (Pluskal et al., 2010) and uploaded to the GNPS-MassIVE mass spectrometry database.

### Riboprobe Preparation for *in situ* Hybridization Histochemistry

Riboprobes targeting the messenger RNA (mRNA) for the rate-limiting enzyme in the synthesis of brain serotonin, tryptophan hydroxylase 2 (*Tph2*) and the sodium-dependent, high-affinity, low-capacity serotonin transporter (solute carrier family 6 member 4, *Slc6a4*) were generated using standard transcription methods as described previously (Day and Akil, 1996). Tryptophan hydroxylase 2 mRNA was detected using a 462 base (1552–2013) antisense riboprobe (from CAL) complementary to the rat mRNA encoding Tph2 [National Center for Biotechnology Information (NCBI) Reference Sequence: NC_005106.4]. Serotonin transporter mRNA was detected using a 491 base (828–1318) antisense riboprobe (originally from Dr. Stanley J. Watson and re-subcloned by Dr. Heidi E. W. Day, Department of Psychology and Neuroscience, University of Colorado Boulder, Boulder, CO, United States) complementary to the mRNA encoding rat Slc6a4 (NCBI Reference Sequence: NC_005109.4).

Riboprobes were radiolabeled via *in vitro* transcription incorporating [^35^S]-UTP. Briefly, a nucleotide mix (1 μl of 10 mM each) of ATP, CTP, and GTP was added to 5 μl 5 × transcription buffer (Cat. no. FP021, Promega, Madison, WI, United States), 2.5 μl 0.1 M dithiothreitol (DTT), and 4 μl sterile MilliQ water. Added to this solution was 1 μl (1 μg) cut DNA (antisense or sense), 1 μl RNase inhibitor (Cat. no. 100000840, RNaseOUT, Invitrogen), 7.5 μl [^35^S]-UTP (1250 Ci/mmol; Cat. no. NEG039H001MC, New England Nuclear-Perkin Elmer, Boston, MA, United States), and 1 μl of the appropriate RNA polymerase (T3 for antisense [Cat. no. P208C] and T7 for sense [Cat. no. P207B], Promega). The mixture was incubated at 37°C for 2 h; template DNA was then removed by digestion with 1 μl RNase-free DNase I (Cat. no. M199A, RQ1 DNase Stop Solution, Promega) for 15 min at 20°C. The probe was purified on G50/50 Sephadex^®^ columns and probe activity (1 μl) was counted in 7 ml ReadySafe^®^ scintillation fluid (Cat. no. p/n484013-ae, Beckman Coulter Inc., Brea, CA, United States) with a Beckman LS 3801 beta counter (Ser. no. 7013835, Beckman Coulter Inc.).

### *In situ* Hybridization Histochemistry

Previously published methods were used for *in situ* hybridization histochemistry (Day and Akil, 1996; Lieb et al., 2019). Briefly, brains were dissected then frozen on dry ice immediately after euthanasia. Brains were sectioned into 12-μm thick sections on a cryostat (Leica CM 1950, Leica Biosystems, Buffalo Grove, IL, United States), in a series of 7 alternate sections throughout the midbrain and pontine raphe nuclei according to a stereotaxic atlas of the mouse brain (Paxinos and Franklin, 2001), thaw-mounted on Histobond^®^ slides (Cat. no. 16,004-406; VWR, Westchester, PA, United States), and stored at −80°C. Due to loss of tissue during sample processing, the sample sizes for *in situ* hybridization histochemistry were as follows: Veh/NLD/SHC, *n* = 7; Veh/NLD/SD, *n* = 8; Veh/CDR/SHC, *n* = 8; Veh/CDR/SD, *n* = 4; *Mv*/NLD/SHC, *n* = 9; *Mv*/NLD/SD, *n* = 6; *Mv*/CDR/SHC, *n* = 7; *Mv*/CDR/SD, *n* = 9.

Tissue sections were fixed in 4% paraformaldehyde for 1 h, acetylated in 0.1 M triethanolamine hydrochloride with 0.25% acetic anhydride for 10 min, and dehydrated through graded alcohols. One slide from each series of 7 alternate slides were used for each gene. Sections were hybridized overnight at 55°C with a [^35^S]-UTP-labeled riboprobe diluted in hybridization buffer containing 50% formamide, 10% dextran sodium sulfate, 2 × saline sodium citrate (SSC), 50 mM phosphate-buffered saline, pH 7.4, 1 × Denhardt’s solution, and 0.1 mg/ml yeast transfer RNA (tRNA). The following day, sections were treated with RNase A, 200 μg/ml at 37°C for 1 h, and washed to a final stringency of 0.1 × SSC at 65°C for 1 h. Dehydrated sections were exposed to x-ray film (BioMax MR, Eastman Kodak, Rochester, NY, United States) for region- and probe-appropriate times (1–3 weeks) prior to film development.

### Imaging and Densitometry of *in situ* Hybridization Histochemistry Autoradiograms

Autoradiographic images of the probe bound to *Tph2* mRNA and *Slc6a4* mRNA, together with ^14^C-labeled standards, were measured using a computer assisted image analysis software system (ImageJ^6^). All measurements were taken by experimenters (KML, LM, and ML) while blinded to the treatment groups. Virtual matrices in the shapes of respective dorsal raphe nucleus (DR) and median raphe nucleus (MnR) subregions were created, overlaid with the image and the “mean gray value (GV) × area” within each matrix was measured, taking into account only the area of the above-threshold signal. During the entire analysis, a constant threshold function was applied, which determined the area that was actually measured within each matrix. Thus, all pixels with a gray density below threshold were automatically excluded. The individual background of each image was measured and subtracted from the mean GV.

Rostrocaudal analysis atlases for *Tph2* and *Slc6a4* expression in the DR and MnR subregions were created by comparing the image of the tissue sections with a stereotaxic mouse brain atlas (Paxinos and Franklin, 2001) and with *Tph2* mRNA expression topography as reported by Lieb et al. (2019). According to Lieb et al. (2019) and Tph protein immunostaining by Abrams et al. (2004), each rostrocaudal level was further divided into respective subregions of the DR. At each rostrocaudal level, the mean GV × area values for the left and right sides of each subdivision were averaged. Overall mRNA expression within the six subregions of the DR were displayed by averaging mean GV × area values across all rostrocaudal levels per treatment group. A total of 14 rostrocaudal levels were studied throughout the forebrain. The subdivisions studied were summarized into the following functional subregions and regions: dorsal raphe nucleus, caudal part (DRC), −4.832 mm to −5.252 mm from bregma; dorsal raphe nucleus, dorsal part (DRD), −4.160 mm to −4.748 mm from bregma; dorsal raphe nucleus, interfascicular part (DRI), −4.580 mm to −5.252 mm from bregma; dorsal raphe nucleus, ventral part (DRV), −4.160 mm to −4.664 mm from bregma; dorsal raphe nucleus, ventrolateral part/ventrolateral periaqueductal gray region (DRVL/VLPAG), −4.496 mm to −4.748 mm from bregma; and MnR, −4.160 mm to −4.748 mm from bregma.

### Statistical Analysis

#### Body Weight, Acute Social Defeat, and Object Location Memory Analyses

Linear mixed model (LMM) analyses fit by restricted maximum likelihood (REML) and Satterthwaite’s estimation of degrees of freedom on an unstructured repeated covariance matrix were conducted separately for: (1) body weight data collected during each cage change cycle throughout the duration of the experiment; (2) individual behaviors in the SD test described in Table 2; and (3) location indices in the OLM test. Mean body weight values, durations of each behavior in the SD test, described in Table 2, and location indices in the OLM test in each treatment group were generated and analyzed using the R package *lmerTest* (Kuznetsova et al., 2017). Extreme statistical outliers were identified using Grubbs’ test for single outliers using a two-sided alpha-threshold of 0.05 (Grubbs, 1969) within each treatment group and were removed from the analyses.

For the five-factor LMM analysis of body weight, *Mv*, CDR, SD, cohort, and time point were used as fixed effects; time point was also used as the repeated effect. For the two-factor LMM analysis of attacking, avoiding, chasing, mounting, scouting, flight, submissive upright posture, attacks received, chasing received, and mounts received, each individual behavior was analyzed separately; *Mv*, CDR, and cohort were used as fixed effects; cohort was also used as the random effect. For the four-factor LMM analysis of location indices, *Mv*, CDR, SD, and cohort were used as fixed effects; cohort was also used as the random effect. Pairwise comparisons were made with Fisher’s least significant difference (LSD) tests. *Post hoc* testing was not conducted at a specific time point if one or more groups contained less than 50% of the full sample size at that time point or endpoint. Additionally, *post hoc* between-subjects analyses were conducted only when the overall LMMs just described yielded significant effects of any factors or interactions among the factors described above. Two-tailed significance was set at *p* < 0.05.

#### 16S rRNA Gene Sequence Data Analysis

Microbial community structure was characterized using measures of alpha-diversity (within-sample) and beta-diversity (between-samples). Metrics of alpha-diversity included number of distinct features to represent species abundance (richness), Shannon’s diversity index to quantify richness and evenness (Shannon et al., 1949), and Faith’s phylogenetic diversity, which measures the total length of branches in a reference phylogenetic tree for all species in a given sample (Faith, 1992).

Beta-diversity was calculated using unweighted UniFrac distances (Lozupone and Knight, 2005; Lozupone et al., 2007, 2011) depicting community-wide differences in microbial composition amongst fecal samples from days –1, 48, and 62. Output distance matrices were ordinated using principal coordinate analyses (PCoA) and visualized using EMPeror (Vázquez-Baeza et al., 2013). Statistical significance of beta-diversity distances between groups was assessed using permutational analyses of variance (PERMANOVA) with 999 Monte Carlo permutations. Alpha-diversity group significance was calculated using non-parametric Kruskal–Wallis *H* tests in QIIME2. LMM analysis fit by REML and Satterthwaite’s estimation of degrees of freedom on an unstructured repeated covariance matrix were conducted on the alpha-diversity metrics generated by QIIME2 and analyzed using the R package *lmerTest* (Kuznetsova et al., 2017) to determine the effects of *Mv*, CDR, SD, time point, and any interactions between the factors just described. Extreme statistical outliers were identified using Grubbs’ test for single outliers using a two-sided alpha-threshold of 0.05 (Grubbs, 1969) within each treatment group and were removed from the analyses.

#### Metabolomics Networking Analysis for Both Fecal and Plasma Metabolomics Datasets and Procrustes Analysis

Molecular networking was performed to identify spectra shared between different sample types and to identify known molecules in the dataset. All annotations are at level 2 (MS^2^) according to the proposed minimum standards in metabolomics. Briefly, multiple spectral alignment of all clustered spectra was performed as in Petras et al. (2017), and all spectra were compared to each other using cosine similarity scoring (Stein and Scott, 1994). Spectrum-spectrum matches with a cosine score higher or equal to 0.7 were connected as “nodes” in a network (Watrous et al., 2012; Wang et al., 2016); if nodes yielded a database library hit during a spectrum library search, they were labeled as specific compounds and nodes around the library hit were assumed to have similar chemical scaffolds. The molecular networking parameters were as follows, in addition to the cosine similarity score cutoff just described: a minimum matched-peak threshold of 4, a minimum cluster size of 2, and a parent and ion tolerance of 0.5 Da. If the delta masses, fragmentation patterns, and chemical formulas of the database hit are carefully interpreted, putative structures of previously unknown spectra can be proposed with a certain confidence.

From these data, a feature table of metabolite absence/presence and relative abundance in each sample was generated from GNPS spectral alignments and downloaded. Similarity of individual mouse metabolomes grouped by *Mv* or Veh treatment was determined using the Canberra-Adkins distance metric, projected with PCoA and visualized with EMPeror through the in-house tool ClusterApp. Molecular networks were visualized and mined using the software program Cytoscape version 3.7.2^7^ (Shannon et al., 2003). Additional visualizations of potential relationships between the fecal 16S rRNA gene sequencing-based microbiome dataset and the fecal LC-MS/MS-based metabolomics dataset were visualized with a Procrustes plot, using unweighted UniFrac and Canberra-Adkins distance matrices respectively.

### *In situ* Hybridization Histochemistry Analysis

*In situ* hybridization histochemistry analysis was conducted separately for *Tph2* and *Slc6a4* mRNA expression using a LMM approach. For analysis of *in situ* hybridization histochemistry data, mean GV × area for each DR subdivision at each rostrocaudal level of the DR in each treatment group were generated and analyzed using the software package IBM SPSS Statistics (version 25.0, SPSS Inc., Chicago, IL, United States). Extreme statistical outliers were identified using Grubbs’ test for single outliers using a two-sided alpha-threshold of 0.05 (Grubbs, 1969) within each *Mv* × CDR × SD treatment group and subregion and were removed from the analysis. For analysis of *Tph2* and *Slc6a4*, a survey of LMM with different covariance structures was performed and the model with the best –2 log-likelihood value for each gene, an information criterion function used for goodness of fit, was selected. For the overall three-factor LMM analysis of *Tph2* and *Slc6a4* mRNA, *Mv* treatment, CDR, SD, cohort, rostrocaudal level, and DR subregion were used as fixed effects; rostrocaudal level was also used as the repeated effect.

Secondary LMMs were run and the best covariance structure was selected using the best –2 log-likelihood value for each individual subregion of the DR, with *Mv* treatment, CDR, SD, and cohort as fixed effects and rostrocaudal level as the repeated effect. Pairwise comparisons were made with Fisher’s LSD tests. As an added criterion in the analysis in the *in situ* hybridization histochemistry analysis, *post hoc* testing was not conducted at a specific rostrocaudal level if one or more groups contained less than 50% of the full sample size at that rostrocaudal level. Additionally, *post hoc* between- and within-subjects analyses were conducted only when overall or secondary LMMs as explained above yielded significant effects of *Mv* treatment, CDR, SD, cohort, interactions among these conditions, or interactions with these factors and rostrocaudal level within the DR subregion or rostrocaudal level. Two-tailed significance was set at *p* < 0.05.

## Results

### CDR Disrupts Diurnal Patterns of Locomotor Activity

Telemetric recordings of LA were assessed to confirm successful implementation of NLD and CDR housing conditions. Representative examples of telemetric recordings of LA illustrating Veh/NLD, *Mv*/NLD, Veh/CDR, and *Mv*/CDR conditions are shown in Supplementary Figure S2. NLD-exposed mice remained stably entrained to the LD cycle throughout the experiment. While CDR-exposed mice primarily exhibited phase delays in response to the LD cycle reversals, full re-entrainment was not always achieved within 7 days (prior to the next LD reversal).

### *Mv* Immunization Reduces Submissive Behavioral Displays in Acute Social Defeat

**FIGURE 2:**
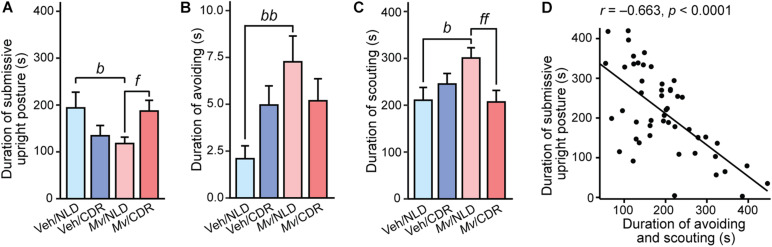
Behavioral responses of experimental mice during the 10-min social defeat test. Individual panels represent means + standard errors of the means of mice from seven independently tested cohorts of mice; **(A)** duration of submissive upright posture, **(B)** duration of avoiding behavior, and **(C)** duration of scouting behavior in s. **(D)** Shows the relationship between the duration of avoiding and scouting (s) and the duration of submissive upright posture (s); circles indicate individual data points. *Post hoc* pairwise comparisons indicated by ^*b*^*p* < 0.05 and ^*bb*^*p* < 0.01, Veh/NLD against *Mv*/NLD group; ^*f*^*p* < 0.05 and ^*ff*^*p* < 0.01, *Mv*/NLD against *Mv*/CDR group. CDR, chronic disruption of rhythms; *Mv*, *Mycobacterium vaccae* NCTC 11659; NLD, normal light:dark cycle; Veh, borate-buffered saline vehicle. For sample sizes of each group, see Supplementary File S1.

Analysis of the duration of submissive upright posture (in s) in the SD test using an overall LMM approach revealed an interaction effect of *Mv* treatment × CDR (*t* = 2.58, *p* = 0.013). Among NLD mice, immunization with *Mv*, relative to Veh-treated controls, decreased the duration of submissive upright posture (*p* = 0.034; Table 3 and Figure 2A). Conversely, among *Mv*-immunized mice, CDR increased submissive upright posture relative to NLD control mice (*p* = 0.044; Table 3 and Figure 2A). Similar analysis on the duration of avoiding revealed an interaction effect of *Mv* treatment × CDR (*t* = –2.05, *p* = 0.047). *Post hoc* pairwise comparisons showed an increase in avoiding among *Mv*-treated NLD mice relative to Veh-treated controls (*p* = 0.003; Table 3 and Figure 2B).

**TABLE 3:** Group means ± standard errors of the means of the durations (s) of each measured behavior in the social defeat test.

**Treatment group**	**Avoiding (s)**	**Scouting (s)**	**Flight (s)**	**Submissive upright posture (s)**	**Attacks received (s)**	**Mounts received (s)**	**Chase received (s)**	**Inactivity (s)**	**Total activity (s)**
Veh/NLD	1.75 ± 0.61	210.66 ± 27.12	17.47 ± 3.14	193.82 ± 33.47	14.90 ± 2.94	46.97 ± 8.70	1.22 ± 0.37	93.66 ± 27.63	580.44 ± 5.44
Veh/CDR	4.58 ± 1.01	245.18 ± 22.55	19.59 ± 2.98	134.26 ± 21.93	13.08 ± 1.88	62.06 ± 15.51	0.78 ± 0.25	70.18 ± 21.65	549.71 ± 26.31
*Mv*/NLD	7.27 ± 1.38^bb^	300.74 ± 21.95^b^	21.54 ± 3.75	117.65 ± 13.75^b^	17.30 ± 2.62	82.41 ± 26.59	1.86 ± 0.80	37.42 ± 10.68	586.18 ± 4.42
*Mv*/CDR	4.81 ± 1.15	206.87 ± 24.76^ff^	21.57 ± 4.26	187.13 ± 22.77^f^	13.40 ± 1.88	56.18 ± 14.65	1.04 ± 0.44	66.73 ± 12.52	557.73 ± 23.81

*Pairwise comparisons indicated by ^*b*^*p* < 0.05 and ^*bb*^*p* < 0.01, Veh/NLD against *Mv*/NLD group; ^*f*^*p* < 0.05 and ^*ff*^*p <* 0.01, *Mv*/NLD against *Mv*/CDR group. *Abbreviations*: CDR, chronic disruption of rhythms; *Mv, Mycobacterium vaccae* NCTC 11659; NLD, normal light:dark cycle; Veh, borate-buffered saline vehicle*

Analysis of the duration of scouting demonstrated an interaction effect of *Mv* treatment × CDR (*t* = –2.56, *p* = 0.014) and a main effect of CDR (*t* = 2.695, *p* = 0.010). *Post hoc* pairwise comparisons showed an increase in duration of scouting among *Mv*-treated NLD mice relative to Veh-treated NLD mice (*p* = 0.014; Table 3 and Figure 2C). Additionally, *Mv*-treated CDR mice showed a decrease in duration of avoiding compared to *Mv*-treated NLD mice (*p* = 0.008; Table 3 and Figure 2C). Duration of avoiding and scouting behaviors combined, two of the behaviors observed most frequently in the SD test, was inversely correlated to the duration of submissive upright posture (Pearson’s *r* = –0.66, *p* < 0.0001; Figure 2D).

### SD Enhances Cognitive Performance in the Object Location Memory Test

**FIGURE 3:**
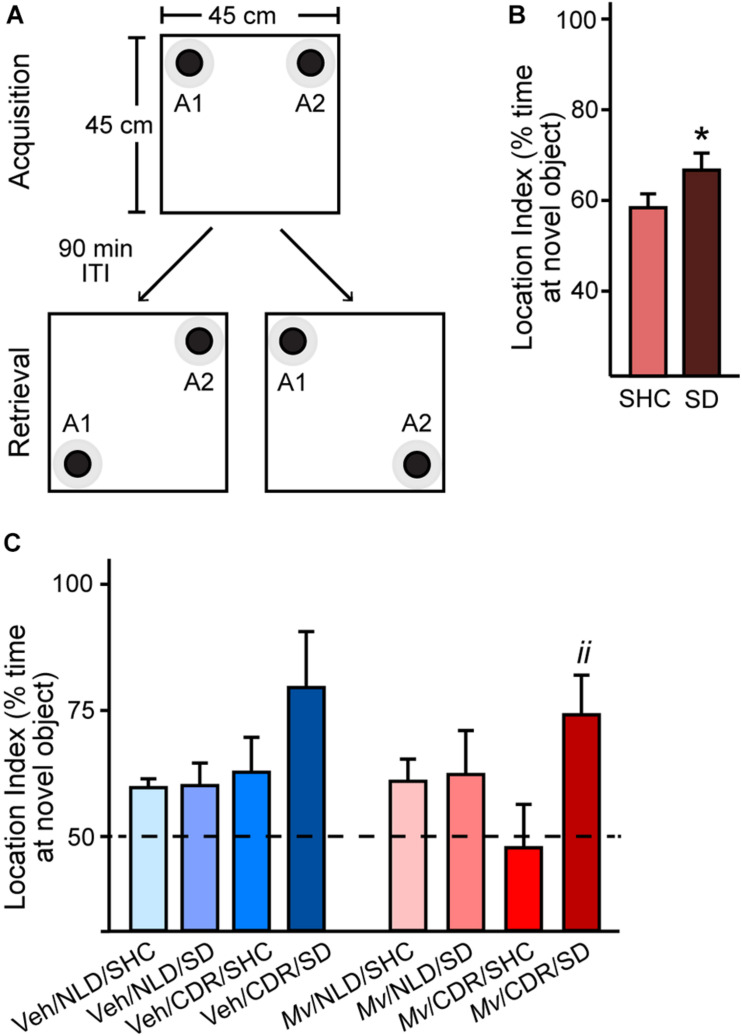
Object location memory test configuration and location indices. **(A)** Arena configuration for the pre-test phase, where black bottlecaps of equal dimensions and coloration (objects A1 and A2; black circles) with defined contact zones (gray circles) were placed in adjacent corners, 2.5 cm away from the walls of the 45 cm × 45 cm × 40 cm arena. Each individual mouse was placed in the center of the arena and permitted to freely explore the arena for 5 min during the pre-test Acquisition phase. The mouse was then removed from the testing arena and returned to its home cage for a 90-min inter-trial interval (ITI) while the testing arena and objects A1 and A2 were cleaned with 70% ethanol and paper towels. The arena was then reconfigured for the test phase (Retrieval) such that either object A1 (lower left) or A2 (lower right) was displaced from its original position, diagonally positioned from the other object in non-adjacent corners. After the 90-min ITI, the mouse was then placed back in the testing arena. Location index data were obtained via Noldus Ethovision XT for **(B)** mice exposed to social defeat (SD) or left undisturbed in single-housed home cage control (SHC) conditions and **(C)** for all treatment groups. Data represent means + standard errors of the means of mice from seven independently tested cohorts of mice. Dashed line represents equal exploration of the displaced object and the unmoved object; bars above this threshold indicate preferential exploration of the displaced object. Pairwise *post hoc* comparisons shown: *p < 0.05, SD against SHC, and ^*ii*^*p* < 0.01, *Mv*/CDR/SD against *Mv*/CDR/SHC. CDR, chronic disruption of rhythms; *Mv*, *Mycobacterium vaccae* NCTC 11659; NLD, normal light:dark condition; SD, social defeat; SHC, single-housed home cage control condition; Veh, borate-buffered saline vehicle. For sample sizes of each group at each time point, see Supplementary File S1.

Analysis of the location indices in the OLM test using an overall LMM approach revealed a main effect of SD (*t* = –2.619, *p* = 0.012; Figures 3A–C). *Post hoc* pairwise comparisons showed an increase in location index among *Mv*-treated CDR mice that underwent SD relative to SHC treatment group controls (*Mv*/CDR/SD: 74.07 + 7.89%, *Mv*/CDR/SHC: 47.74 + 8.61%, *p* = 0.008; Figure 3C).

### *Mv* Stabilizes CDR-Induced Decreases in Microbial Alpha- and Beta-Diversity

**FIGURE 4:**
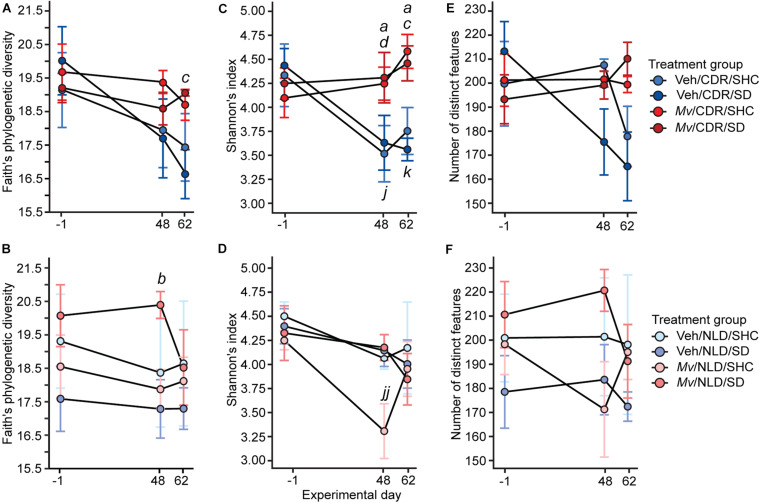
Within-subject alpha diversity of samples shown separately for chronic disruption of rhythms (CDR) conditions and normal light:dark conditions (NLD), as measured by **(A,B)** Faith’s phylogenetic diversity, **(C,D)** Shannon’s index, and **(E,F)** number of distinct features over time. Data represent treatment group means ± standard errors of the means of alpha diversity in fecal samples from days –1, 48, and 62 of mice in five independently tested cohorts of mice. Analyses were performed on 16 small subunit ribosomal RNA (16S rRNA) gene V4 amplicon data at a rarefaction depth of 15,000 reads per sample. 16S rRNA, 16 small subunit ribosomal RNA; CDR, chronic disruption of rhythms; *Mv*, *Mycobacterium vaccae* NCTC 11659; NLD, normal light:dark conditions; SD, social defeat; SHC, single-housed home cage control condition; Veh, borate-buffered saline vehicle. For sample sizes of each group at each time point, see Supplementary File S1; for definitions of symbols used to indicate significant *post hoc* pairwise comparisons, see Table 4.

**FIGURE 5:**
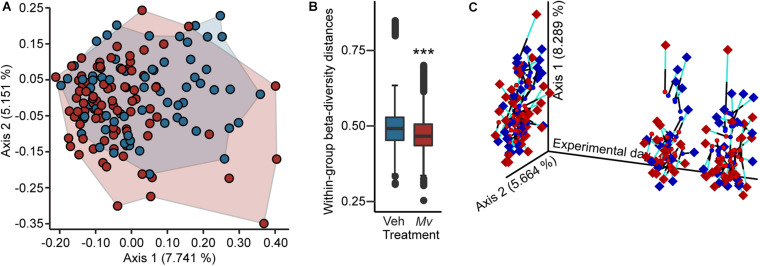
Beta diversity and Procrustes analyses of gut microbiomes. **(A)** Principal coordinates analysis (PCoA) of microbial community-wide beta diversities of mice from five independently tested cohorts of mice using Unweighted UniFrac, grouped by treatment (Veh, blue; *Mv*, red). PCoA axes 1 and 2 explain 12.9% of the variation. **(B)** Within-group beta diversity distances among individual samples (Veh, blue; *Mv*, red); ***p < 0.0001, Kruskal–Wallis *H* test. **(C)** Procrustes analysis plot produced from the superimposition of the PCoA of the 16 small subunit ribosomal RNA (16S rRNA) gene V4 amplicon data (circles) and the LC-MS/MS-based metabolomics dataset (diamonds). Longer lines on Procrustes plots indicate greater within-subjects dissimilarity of the microbiome and metabolome. PCoA axes 1 and 2 explain 14.0% of the variation observed in the data. For sample sizes of each group at each time point, see Supplementary File S1. 16S rRNA, 16 small subunit ribosomal RNA; *Mv*, *Mycobacterium vaccae* NCTC 11659; PCoA, principal coordinates analysis; Veh, borate-buffered saline vehicle.

Analysis of the microbial alpha-diversity in the 16S rRNA gene data demonstrated that CDR reduced microbial diversity between days –1 and 48 and that *Mv* treatment prior to day –1 ameliorated this reduction. Using an overall LMM approach, separate analyses of each alpha-diversity metric previously described revealed a main effect of *Mv* treatment [*F*(1, 124) = 4.75, *p* = 0.031; Figures 4A,B] in Faith’s phylogenetic diversity, interactions of *Mv* treatment × CDR × time point [*F*(2, 127) = 3.71, *p* = 0.027; Figures 4C,D] and *Mv* treatment × CDR [*F*(1, 127) = 10.71, *p* = 0.001; Figures 4C,D] in Shannon’s diversity index, and interactions of *Mv* treatment × SD [*F*(1, 126) = 4.49, *p* = 0.036; Figures 4E,F] in the number of distinct features. Analysis of microbial beta-diversity using PERMANOVAs revealed community-level separation between *Mv* and Veh groups using unweighted UniFrac (pseudo-*F* = 2.28, *p* = 0.001; 999 Monte Carlo permutations; Figure 5A). Analysis of the unweighted UniFrac distance matrix also demonstrated that the within-group distances of samples in the *Mv* group were smaller than those in the Veh group (*p* < 0.0001, Kruskal–Wallis *H* test; Figure 5B).

**TABLE 4:** Definitions of symbols used to indicate significant *post hoc* pairwise comparisons in figures.

**Symbol**	**Pairwise comparison**	**Effect**
*	*Mv*/NLD/SHC versus Veh/NLD/SHC unless otherwise indicated in the figure legend	*Mv* effect among NLD/SHC mice unless otherwise indicated in the figure legend
a	*Mv*/CDR/SHC versus Veh/CDR/SHC	*Mv* effect among CDR/SHC mice
b	*Mv*/NLD/SD versus Veh/NLD/SD	*Mv* effect among NLD/SD mice
c	*Mv*/CDR/SD versus Veh/CDR/SD	*Mv* effect among CDR/SD mice
§	Veh/CDR/SHC versus Veh/NLD/SHC	CDR effect among Veh/SHC mice
d	*Mv*/CDR/SHC versus *Mv*/NLD/SHC	CDR effect among *Mv*/SHC mice
e	Veh/CDR/SD versus Veh/NLD/SD	CDR effect among Veh/SD mice
f	*Mv*/CDR/SD versus *Mv*/NLD/SD	CDR effect among *Mv*/SD mice
#	Veh/NLD/SD versus Veh/NLD/SHC	SD effect among Veh/NLD mice
g	*Mv*/NLD/SD versus *Mv*/NLD/SHC	SD effect among *Mv*/NLD mice
h	Veh/CDR/SD versus Veh/CDR/SHC	SD effect among Veh/CDR mice
i	*Mv*/CDR/SD versus *Mv*/CDR/SHC	SD effect among *Mv*/CDR mice
j	Within-group, day 48 against day –1	Within-group time effect
k	Within-group, day 62 against day –1	Within-group time effect

*CDR, chronic disruption of rhythms; *Mv, Mycobacterium vaccae* NCTC 11659; NLD, normal light:dark cycle; SD, social defeat; SHC, single-housed home cage control condition; Veh, borate-buffered saline vehicle.*

### Procrustes Analysis

Procrustes analysis revealed concordance among fecal microbiomes and fecal metabolomes of individual mice from samples taken on days –1, 48, and 62 (Figure 5C).

### *Mv* Increases the Production of Lysophospholipids in the Plasma Metabolome

**FIGURE 6:**
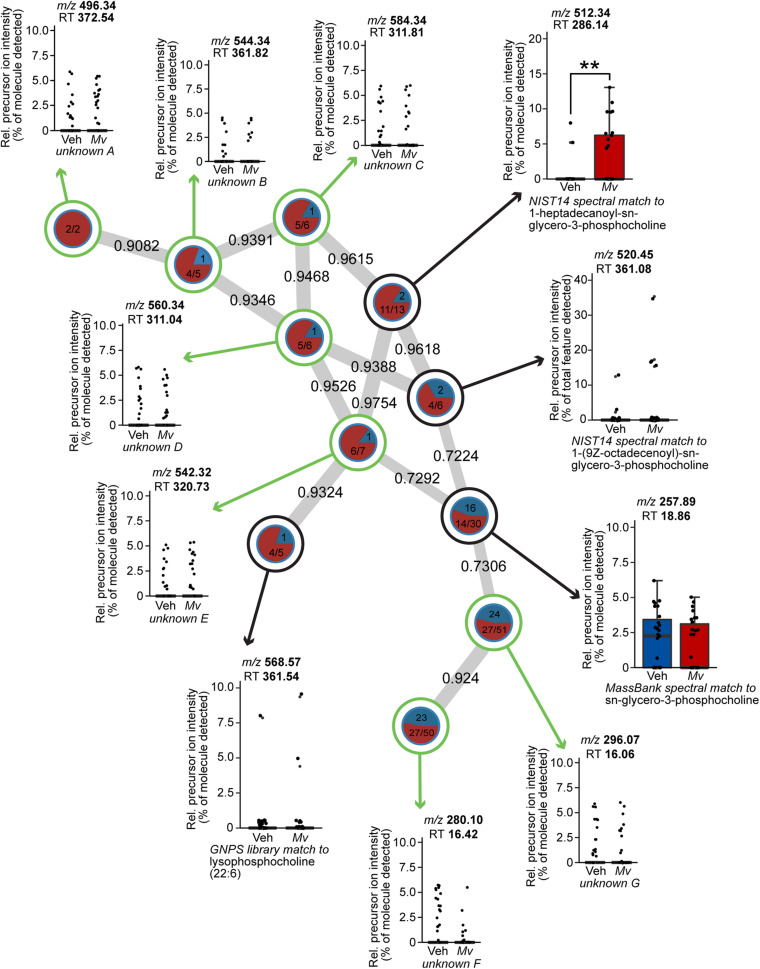
Spectral network of a molecular family of lysophospholipids present in the untargeted plasma metabolomics dataset of mice from six independently tested cohorts of mice. Nodes from solvent blanks, standards, and controls were subtracted. Nodes are labeled according to treatment group origin (Veh, blue; *Mv*, red) if spectra came exclusively from a single group, as in unknown A, and are split into labeled frequency pie charts if spectra above the lower limit of detection came from both groups, as in unknown B. Nodes without a library hit are circled in green, identified according to precursor mass-to-charge ratio (*m/*z) and retention time (RT) in s, and annotated as unknowns A–G. Nodes with a library hit are circled in black and identified according to spectral database and library compound name, in addition to *m/z* and RT information as above. For sample sizes of each group, see Supplementary File S1. GNPS, Global Natural Products Social Molecular Networking; *Mv, Mycobacterium vaccae* NCTC 11659; *m/z*, mass-to-charge ratio; NIST, National Institutes of Standards and Technology; Rel., relative; RT, retention time; Veh, borate-buffered saline vehicle.

Node-centric Cytoscape molecular networking analysis of the plasma metabolomics dataset demonstrated that a single subnetwork of molecular features in the lysophosphatidylcholine (lysoPC) family had an uneven frequency distribution above the minimum detectable threshold in *Mv*-treated mice relative to Veh-treated mice (Figure 6). Unpaired *t*-tests of the relative precursor ion intensity values obtained from GNPS demonstrated that *Mv-*treated animals had greater relative precursor ion intensities of 1-heptadecanoyl-sn-glycero-3-phosphocholine, also commonly known as lysoPC (17:0), compared to Veh-treated animals (*p* = 0.0098, Figure 6).

### *Mv* Stabilizes Serotonergic Gene Expression in the “Two Hit” Model

#### *In situ* Hybridization Histochemistry of *Tph2*

**FIGURE 7:**
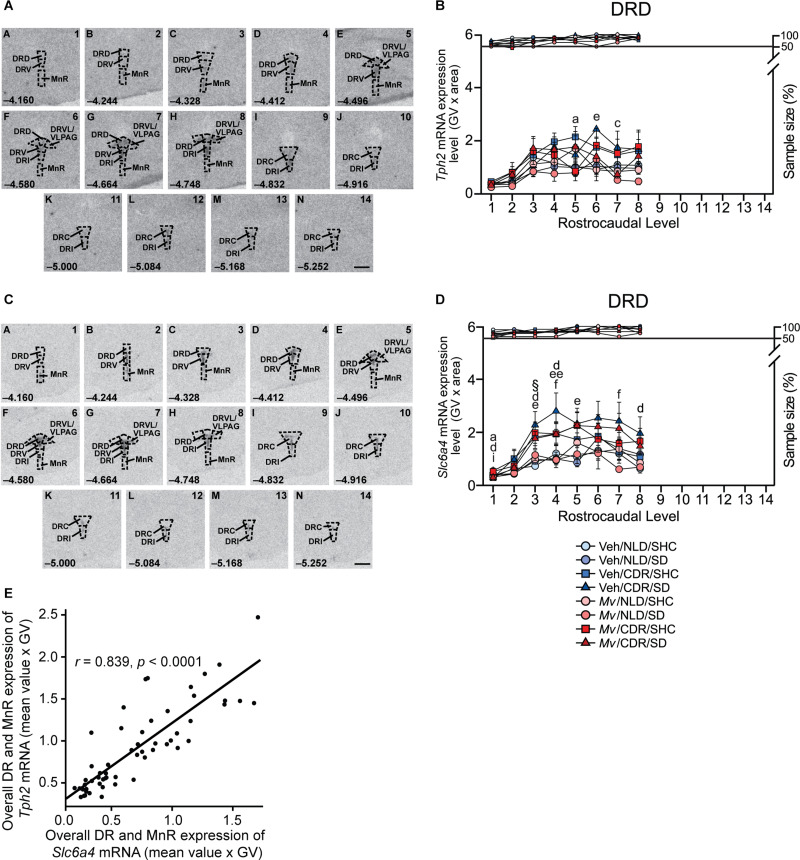
*In situ* hybridization histochemistry-based analysis of tryptophan hydroxylase 2 (*Tph2*) and high-affinity, low-capacity sodium-dependent serotonin transporter (*Slc6a4*) mRNA expression. Atlases **(A,C)** based on autoradiographic images of **(A)** *Tph2*, and **(C)** *Slc6a4* mRNA expression in the midbrain and pontine raphe complex (84 μm intervals) from a representative adult male mouse in this study, used for analysis of subregions of the dorsal raphe nucleus (DR) and median raphe nucleus (MnR) with a high level of neuroanatomical resolution. The levels chosen for analysis ranged from (A) –4.160 mm bregma through (N) –5.252 mm bregma. Dashed lines delineate different subdivisions of the DR and MnR analyzed in this study, based on a stereotaxic atlas of the mouse brain (Paxinos and Franklin, 2001). Numbers in the lower left of each panel indicate the rostrocaudal coordinates relative to bregma in mm. Numbers in the upper right of each panel correspond to the *x*-axis values in graphical representation of the data. Scale bar, 1 mm. Effects of immunization with *Mycobacterium vaccae* NCTC 11659 (*Mv)*, exposure to chronic disruption of rhythms (CDR), and exposure to social defeat (SD) on **(B)** *Tph2* mRNA expression within the dorsal raphe nucleus, dorsal part (DRD) and **(D)** on *Slc6a4* mRNA expression within the DRD. Data represent treatment group means + standard errors of the means of mice from six independently tested cohorts of mice at each rostrocaudal level throughout the DRD. *Post hoc* testing was not conducted when one or more groups contained less than 50% of the full sample size, indicated by the right y-axis in **(B)** and **(D)**. **(E)** *Tph2* and *Slc6a4* mRNA expression throughout the rostrocaudal extent of the DR and MnR are highly correlated (Pearson’s *r* = 0.839, *p* < 0.0001). For sample sizes of each group, see Supplementary File S1. For definitions of symbols used to indicate significant *post hoc* pairwise comparisons, see Table 4. CDR, chronic disruption of rhythms; DR, dorsal raphe nucleus; DRC, dorsal raphe nucleus, caudal part; DRD, dorsal raphe nucleus, dorsal part; DRI, dorsal raphe nucleus, interfascicular part; DRV, dorsal raphe nucleus, ventral part; DRVL/VLPAG, dorsal raphe nucleus, ventrolateral part/ventrolateral periaqueductal gray; MnR, median raphe nucleus; GV, gray value; mRNA, messenger RNA; *Mv*, *Mycobacterium vaccae* NCTC 11659; SD, social defeat; SHC, single-housed home cage control condition; *Slc6a4*, solute carrier family 6 member 4 (high-affinity, low-capacity sodium-dependent serotonin transporter); *Tph2*, tryptophan hydroxylase 2.

Analysis of the *Tph2* mRNA expression in the DR using an overall LMM approach revealed an interaction effect of *M*v treatment × CDR × rostrocaudal level [*F*(13, 200.58) = 3.47, *p* < 0.001; Supplementary Table S1] and a main effect of SD [*F*(1, 289.82) = 4.26, *p* = 0.040; Supplementary Table S1]. Based on the overall LMM analysis, secondary LMMs were used to determine the effects of *Mv*, CDR, and SD on *Tph2* mRNA expression within each subregion of the DR and MnR (Supplementary Table S2). Secondary LMMs revealed a main effect of CDR on the levels of *Tph2* mRNA expression within the DRD subregion [*F*(1, 43.39) = 6.19, *p* = 0.017; Supplementary Table S2, Figures 7A,B, and Supplementary Figures S3A, S4A). An interaction effect of *Mv* treatment × CDR × rostrocaudal level was evident in the DRV [*F*(6, 37.56) = 3.02, *p* = 0.016; Supplementary Table S2 and Supplementary Figures S3B, S4B). A main effect of CDR on the levels of *Tph2* mRNA expression within the DRVL subregion was also evident [*F*(1, 44.11) = 5.43, *p* = 0.024; Supplementary Table S2 and Supplementary Figures S3C, S4C). Interaction effects of *Mv* treatment × CDR × rostrocaudal level were evident in the DRC [*F*(5, 27.88) = 3.28, *p* = 0.019; Supplementary Table S2 and Supplementary Figures S3D, S4D] and in the DRI [*F*(8, 32.84) = 2.78, *p* = 0.018; Supplementary Table S2 and Supplementary Figures S3E, S4E]. There were no effects of *Mv*, CDR, SD, rostrocaudal level, or interactions among these factors in the MnR.

Based on *post hoc* pairwise comparisons, the most consistent effect was an effect of CDR, relative to NLD conditions, to increase *Tph2* mRNA expression among Veh/SD mice, an effect that was observed in the DRD, DRV, and DRI (Figure 7B and Supplementary Figures S3, S4). This effect of CDR to increase *Tph2* mRNA expression was also observed among *Mv*/SD mice, but in different subregions, namely the DRVL/VLPAG and MnR (Supplementary Figures S3, S4). These data suggest that immunization with *Mv* stabilized *Tph2* mRNA expression under CDR/SD conditions in the DRD, DRV, and DRI, but increased *Tph2* mRNA expression in the DRVL/VLPAG and MnR (Supplementary Figure S3), two structures implicated in enhanced stress coping (Paul and Lowry, 2013). This is supported by effects of *Mv* to decrease *Tph2* mRNA expression among CDR/SD mice in the DRD, an anxiety-related subregion of the DR (Figure 7B and Supplementary Figure S3A, S4A) (Lowry et al., 2008; Hassell et al., 2017).

Exposure to CDR in Veh/SHC mice also increased *Tph2* mRNA expression, in this case in the DRVL/VLPAG, DRC, DRI, and overall DR and MnR (Supplementary Figures S3, S4), suggesting that CDR increased *Tph2* mRNA expression with or without subsequent exposure to SD.

Immunization with *Mv* also decreased *Tph2* mRNA expression among CDR/SHC mice in the DRD, DRV, DRVL/VLPAG, and DRI, again suggesting that immunization with *Mv* stabilized *Tph2* mRNA expression under CDR conditions. The ability of *Mv* to stabilize *Tph2* mRNA expression in the DRD, DRV, and DRI in mice exposed to CDR was observed both in the presence and absence of subsequent exposure to SD.

#### *In situ* Hybridization Histochemistry of *Slc6a4*

Analysis of *Slc6a4* mRNA expression using LMM analysis revealed an interaction effect of *Mv* treatment × CDR × SD × rostrocaudal level [*F*(26, 212.47) = 2.06, *p* = 0.003; Supplementary Table S3]. Based on the overall LMM analysis, secondary LMMs were used to determine the effects of *Mv*, CDR, and SD on *Slc6a4* mRNA expression within each subregion of the DR and MnR (Supplementary Table S4). Interaction effects of CDR × rostrocaudal level were evident in the DRD [*F*(7, 40.46) = 5.48, *p* < 0.001; Supplementary Table S4, Figures 7C,D, and Supplementary Figures S5A, S6A) and DRV [*F*(6, 40.25) = 3.38, *p* = 0.009; Supplementary Figures S5B, S6B). Interaction effects of *Mv* treatment × CDR × SD were evident in the DRC [*F*(1, 43.67) = 5.71, *p* = 0.021; Supplementary Table S4 and Supplementary Figures S5D, S6D]. Interaction effects of *Mv* treatment × CDR × SD × rostrocaudal level were evident in the DRI [*F*(16, 37.43) = 2.95, *p* = 0.003; Supplementary Table S4 and Supplementary Figures S5E, S6E]. Interaction effects of *Mv* treatment × rostrocaudal level [*F*(7, 35.56) = 3.03, *p* = 0.013; Supplementary Table S4 and Supplementary Figures S5F, S6F] and main effects of CDR [*F*(1, 41.04) = 9.00, *p* = 0.005] were also evident in the MnR.

Based on *post hoc* pairwise comparisons, the most consistent effect was an effect of CDR, relative to NLD conditions, to increase *Slc6a4* mRNA expression among Veh/SD mice, an effect that was observed in the DRD, DRV, DRC, DRI, and MnR, as well as in the overall DR and MnR (Supplementary Figures S5, S6). This effect of CDR to increase *Slc6a4* mRNA expression was rarely observed among Mv/SD mice, and not at all in the DRV, DRC, DRI, or overall DR and MnR (Supplementary Figures S5, S6), suggesting that immunization with *Mv* stabilized *Slc6a4* mRNA expression under CDR/SD conditions. This is supported by effects of *Mv* to decrease *Slc6a4* mRNA expression among CDR/SD mice, an effect that was evident in the DRC, DRI, and overall DR and MnR (Supplementary Figure S5,S6). *Slc6a4* mRNA expression and *Tph2* mRNA expression in the overall DR and MnR were highly correlated (Figure 7E and Supplementary Figures S3G, S4G, S5G, S6G).

## Discussion

*Mycobacterium vaccae* NCTC 11659, a soil-derived bacterium with anti-inflammatory and immunoregulatory properties, promoted stress resilience in a “two hit” stressor model consisting of CDR followed by SD. A number of observations supported the conclusion that immunization with *M. vaccae* altered microbiome-gut-brain axis signaling, leading to increased stress resilience. Among mice exposed to NLD control conditions, immunization with *M. vaccae* shifted behavioral responses during SD toward an increase in proactive coping behavioral displays and toward a decrease in stereotyped displays of submission during the establishment of social dominant/subordinate relationships. Interestingly, this effect of *M. vaccae* was absent in mice exposed to CDR, suggesting that stress resilience effects of *M. vaccae* to promote proactive coping behavioral responses can be disrupted, through unknown mechanisms, by CDR. Contrary to our expectations, SD exposure increased, rather than decreased, cognitive performance in the OLM test, an observation that is consistent with previous studies demonstrating that acute exposure to stress or acute injections of glucocorticoids can enhance memory consolidation (for review, see Hassell et al., 2019b); however, this effect was only observed in *M. vaccae*-immunized mice that underwent CDR. Immunization with *M. vaccae* prevented stress-induced decreases in alpha diversity of the gut microbiome following CDR and stabilized the community-wide gut microbiome as measured by within-group beta-diversity. Evidence suggests that immunization with *M. vaccae* increased the relative abundance of a family of lysophospholipids, including 1-heptadecanoyl-sn-glycero-3-phosphocholine. Given that lysoPCs have a role in lipid signaling by acting exclusively on lysophospholipid receptors (LPL-Rs), lysoPCs emerge as interesting candidates for mediating some of the physiological and behavioral responses that have been described following immunization with *M. vaccae*. Finally, immunization with *M. vaccae* prevented stress-induced increases in expression of serotonergic genes, including *Tph2* and *Slc6a4*, in the DRD, a subregion of the dorsal raphe nucleus that has been well-characterized as responsive to stress- and anxiety-provoking stimuli.

### Effects of *M. vaccae* NCTC 11659 on Behavioral Coping Strategies During Social Defeat

Among mice exposed to NLD conditions, immunization with *M. vaccae*, with the final immunization occurring on day 27 (4 weeks before behavioral testing in the SD test), shifted behavioral responses during SD toward active avoiding and scouting, with concomitant decreased time spent in submissive upright postures. This finding is consistent with previous studies in which immunization with *M. vaccae* decreased submissive upright postures in subordinate mice in the chronic subordinate colony housing (CSC) model 1, 2, or 4 weeks following the final immunization with *M. vaccae* (Reber et al., 2016a,b). In the current study, mice immunized with *M. vaccae* spent more time engaged in behaviors that involved actively avoiding the dominant resident aggressor, including avoiding and scouting. Indeed, mice that spent more time engaged in avoiding and scouting behaviors also spent less time in the submissive upright posture; therefore, increased avoiding and scouting may represent a proactive behavioral strategy to obviate direct confrontation with the dominant resident aggressor and submission. Previous studies have shown that a reactive emotional coping strategy during social defeat predicts vulnerability to subsequent development of anxiety- and depressive-like behavioral responses (Koolhaas et al., 1999; Veenema et al., 2007; Wood et al., 2010; Wood and Bhatnagar, 2015). Thus, a decrease in the duration of submissive behaviors in *M. vaccae*-immunized mice is consistent with a stress-resilient behavioral phenotype.

The mechanisms through which immunization with *M. vaccae* NCTC 11659 promotes a more active behavioral coping strategy during social defeat is not clear. In previous studies by Reber and colleagues (Reber et al., 2016b), depletion of Treg did not affect the frequency of submissive upright postures among mice immunized with *M. vaccae* at any point during the CSC procedure (Reber et al., 2016b). This result might be expected if the effects of *M. vaccae* on upright submissive postures are dependent on myeloid-derived cells, rather than Treg. Previous studies have shown that a decreased plasma IL-6 response to repeated social defeat, assessed either 20 min or 48 h post-defeat, predicts stress resilience in mice (Hodes et al., 2014). Mice prone to the development of a stress-susceptible phenotype also had higher pre-stress levels of circulating leukocytes, predominantly due to monocyte populations, which produced greater amounts of IL-6 when stimulated *ex vivo* with lipopolysaccharide (LPS) (Hodes et al., 2014). Several observations support the conclusion that IL-6 secretion from peripheral myeloid cells are causal in determining stress resilience versus susceptibility in the repeated social defeat model: transplantation of IL-6–deficient bone marrow-derived hematopoietic progenitors promotes stress resilience in the recipient; transplantation of bone marrow-derived hematopoietic progenitor cells from susceptible donors promotes susceptibility to psychosocial stress in the recipient; and inactivation of peripheral IL-6 using monoclonal antibodies prevents the maladaptive behavioral coping responses in susceptible mice (Hodes et al., 2014). Consistent with these findings, rats with fearful temperament respond to low/moderate doses of LPS with exaggerated IL-6 release (Michael et al., 2020). Thus, molecular constituents of *M. vaccae* that decrease IL-6 secretion from peripheral myeloid-derived cells may mediate its stress resilience effects as measured by behavioral responses during SD. In this context, the novel *M. vaccae*-derived lipid, 10(*Z*)-hexadecenoic acid, which, through upregulation of peroxisome proliferator-activated receptor alpha (PPARα) signaling, decreases IL-6 mRNA and protein expression following LPS-challenge in peritoneal macrophages (Smith et al., 2019), is an interesting candidate for mediating these stress resilience effects of *M. vaccae* in the SD paradigm.

The effect of *M. vaccae* to shift behavioral responses toward a more proactive coping response was absent in mice exposed to CDR, suggesting that *M. vaccae*’s stress resilience effects may be disrupted, through unknown mechanisms, by CDR. By design, we chose to test mice in the SD paradigm five days after the final 12-h LD transition so that mice would not be fully entrained to the new light cycle at the time of testing (i.e., to maximize the severity of the “two hit” stressor model). Based on previous studies, mice exposed to CDR induced by weekly reversals of the LD cycle exhibited an exaggerated microbiome shift in response to a high-fat, high-sugar “Western” diet (Voigt et al., 2014), and CDR induced by reversals of the LD cycle every five days increased mortality in response to dextran sodium sulfate (DSS)-induced colitis (Preuss et al., 2008). Mice exposed to CDR in the form of a weekly 6-h phase advance of the LD cycle for 4 weeks respond to the shift with sustained hypothermia and a five-fold increase in endotoxin challenge-induced mortality (Castanon-Cervantes et al., 2010). Furthermore, in the same model of CDR, LPS-induced secretion of IL-6 in whole blood is increased at all points of the circadian cycle, relative to NLD conditions (Adams et al., 2013; Brager et al., 2013). Thus, it is possible that CDR’s proinflammatory effects 4 weeks following the final immunization with *M. vaccae* eclipsed the anti-inflammatory and stress resilience effects induced by *M. vaccae* during SD. It remains to be determined if immunization with *M. vaccae* after CDR could restore stress resilience-associated effects observed during subsequent SD.

### Effects of *M. vaccae* NCTC 11659 on the Gut Microbiome Diversity and Community Structure

Overall, the current study supports the hypothesis that immunization with *M. vaccae* alters microbiome-gut-brain axis signaling, resulting in increased stress resilience. Immunization with *M. vaccae* prevented the effects of the “two hit” stressor model to decrease alpha diversity of the gut microbiome; this effect was most evident using Shannon’s diversity index, a measure of both richness and evenness. These data are in alignment with previous studies in which *M. vaccae* prevented stress-induced decreases in alpha diversity in the CSC model of chronic psychosocial stress (Reber et al., 2016b). Also consistent with previous studies (Reber et al., 2016b), immunization with *M. vaccae* decreased within-group beta diversity measures (i.e., *M. vaccae* vs. *M. vaccae* beta diversity distances were smaller than Veh vs. Veh beta diversity distances). This may be due to the ability of *M. vaccae* to limit inflammation in the host, and thus decrease stochastic inter-individual variability in gut microbiome composition attributed to host inflammation (Zhang et al., 2015).

### Effects of *M. vaccae* NCTC 11659 on the Host Plasma Metabolome

Network-based analysis of the host plasma metabolome suggested that immunization with *M. vaccae* increased the relative abundance of a molecular family of lysoPCs. Immunization with *M. vaccae* specifically increased the relative abundance of 1-heptadecanoyl-sn-glycero-3-phosphocholine in plasma, consistent with a previous study in humans showing that individuals with active *Mycobacterium tuberculosis* infections had increased abundance of three metabolites in plasma relative to healthy controls: phosphatidylglycerol (16:0), lysophosphatidylinositol (18:0), and acylphosphatidylinositol mannoside (56:1) (Collins et al., 2018). LysoPCs have a role in lipid signaling by acting on lysophospholipid receptors such as GPR4, G2A, GRP119, and GPR5(w), members of the G protein-coupled receptor family of integral membrane proteins, although this is very likely to be an incomplete list of signaling mechanisms through which lysoPCs could impact host physiology and behavior (Drzazga et al., 2014; Gendaszewska-Darmach and Drzazga, 2014). LysoPCs may alter innate immune function; for example, G2A receptor deficiency in mice promotes macrophage activation, including increased IL-6 secretion (Bolick et al., 2009). LysoPCs also function to enhance brain uptake of beneficial fatty acids, such as the omega-3 fatty acid docosahexaenoic acid (DHA), an effect that has been associated with improved spatial learning and memory in mice (Sugasini and Subbaiah, 2017; Sugasini et al., 2017). Transport of DHA in lysoPC-conjugated form is the brain’s preferred method of DHA uptake; this method is intracellularly mediated by the major facilitator superfamily domain-containing protein 2A (Mfsd2a) and is not dependent on blood-brain barrier integrity or tight junctions (Nguyen et al., 2014). Together, these data are consistent with the hypothesis that increased plasma concentrations of lysoPCs may be biomarkers of exposures to mycobacterial strains such as *M. vaccae*. Furthermore, lysoPCs emerge as interesting candidates for mediating some of the physiological and behavioral responses that have been described following immunization with *M. vaccae* (Lowry et al., 2007, 2016; Reber et al., 2016b; Bowers et al., 2017, 2019; Fox et al., 2017; Fonken et al., 2018; Frank et al., 2018; Siebler et al., 2018; Amoroso et al., 2019a,b; Hassell et al., 2019a; Loupy et al., 2019).

### Effects of *M. vaccae* NCTC 11659 on the Host Brainstem Serotonergic System

Consistent with the hypothesis that immunization with *M. vaccae* altered microbiome-gut-brain axis signaling relevant to stress resilience, *M. vaccae* altered serotonergic gene expression in the DR. Specifically, mice exposed to the “two hit” stressor (i.e., CDR/SD), relative to mice exposed to SD alone (i.e., NLD/SD), responded with increased *Tph2* and *Slc6a4* mRNA expression in the dorsal part of the DR (DRD). This effect was prevented by prior immunization with *M. vaccae*. The mid-rostrocaudal DRD is considered a stress- and anxiety-responsive subregion of the DR (Lowry et al., 2005, 2008; Donner et al., 2016, 2018, 2020; Hassell et al., 2017), and previous studies have demonstrated that single or “two hit” stressors increase both *Tph2* and *Slc6a4* mRNA expression in this region (Gardner et al., 2009a,b; Donner et al., 2018). Also of interest, mice exposed to the “two hit” stressor, relative to mice exposed to SD alone, responded with increased *Tph2* and *Slc6a4* mRNA expression in the interfascicular part of the DR (DRI). This effect is consistent with previous studies demonstrating that depletion of Treg (associated with an increase in inflammatory signaling) also increases *Tph2* mRNA expression in the DRI (Reber et al., 2016b). Importantly, immunization with *M. vaccae* prevented the effects of the “two hit” stressor on *Tph2* and *Slc6a4* mRNA expression in the DRI. Together, these data further support the hypothesis that immunization with *M. vaccae* NCTC 11659 increases stress resilience, in part through altered microbiome-gut-brain axis signaling. Further studies are required to fully elucidate the mechanisms involved.

### Potential Role of T Effector Cells and Treg in the Effects of *M. vaccae* NCTC 11659, CDR, and SD

Together with previous studies, these data are consistent with the hypothesis that circadian misalignment induces a chronic low-grade inflammation that interferes with stress resilience effects of *M. vaccae* NCTC 11659, as measured during acute SD. The chronic low-grade inflammation induced by circadian misalignment may involve changes in adaptive immunity. Previous studies in mice have shown that long-term circadian misalignment in which mice are unable to resynchronize their activity to the shifted light-dark cycle, and therefore experience continuous non-adjustive and disturbed behavioral rhythms, increases T cell senescence and enriches Th17 effector cell populations (Inokawa et al., 2020). Indeed, long-term circadian misalignment increases the mortality rate of mice by 20-fold, in association with evidence of an enhanced systemic inflammatory response (Inokawa et al., 2020). Specifically, mesenteric lymph node cells from mice exposed to chronic circadian misalignment show increased differentiation of IL-17A^+^ Th17 cells (Inokawa et al., 2020). Although effects of acute SD on adaptive immunity are not well characterized, exposure of male C57BL/6J mice to repeated SD by a larger, dominant male CD1 mouse results in increases in splenic IL-17-producing CD4^+^ and CD8^+^ T cells and reduced Treg in stress-susceptible mice, relative to control mice, as defined by the social interaction ratio in a social interaction test (Ambrée et al., 2019). These data are in turn consistent with previous studies demonstrating that development of learned helplessness in mice is dependent on increased Th17 responses, through RORγt- and IL-17A-dependent mechanisms (Beurel et al., 2013). In summary, data suggest that circadian misalignment, which persisted for 4 weeks following the final immunization with *M. vaccae* NCTC 11659 in our study, can increase Th17 differentiation, which can in turn bias toward a stress susceptible phenotype.

Given that inoculation with single strains of bacteria can restore Treg to normal levels in germ-free mice (Sefik et al., 2015), and efficiently induce RORγt^+^ Treg (Ohnmacht et al., 2015), microbiome-based interventions have potential to promote immunoregulation and suppress chronic low-grade inflammation induced by circadian misalignment and psychosocial stress-induced inflammation that drives stress susceptibility. Subordinate status in the CSC mouse model is associated with decreased numbers of Treg and increased T cell effector function (Schmidt et al., 2010; Langgartner et al., 2015). In contrast, consistent with previous studies in mice (Zuany-Amorim et al., 2002b), immunization with *M. vaccae* NCTC 11659 increases Treg isolated from mesenteric lymph nodes, and depletion of CD4^+^CD25^+^Foxp3^+^ Treg in mice exposed to psychosocial stress in the CSC paradigm prevents the protective effects of *M. vaccae* NCTC 11659 on spontaneous colitis and anxiety-like defensive behavioral responses (Reber et al., 2016b). The long-term, Treg-dependent protection from stress-induced exaggeration of spontaneous colitis and anxiety-like defensive behavioral responses in the CSC paradigm (measured 4 weeks after the final immunization with heat-killed *M. vaccae* NCTC 11659) are consistent with the long-term, Treg-dependent protection from allergic airway inflammation in a murine model of allergic asthma (Zuany-Amorim et al., 2002a,b). Finally, the time course of protective effects of *M. vaccae* NCTC 11659, in the context of spontaneous colitis and anxiety-like defensive behavioral responses (Reber et al., 2016b), are consistent with the time course of induction of Treg following infection of mice with a live strain of *M. vaccae* isolated from bovine submaxillary lymph nodes, in which CD4^+^CD25^+^Foxp3^+^ Treg are elevated within 1 week of infection, reach maximal levels 3 or 4 weeks following infection, and remain elevated until at least 8 weeks following infection (Zhang et al., 2016).

## Conclusion

Immunization with a heat-killed preparation of *M. vaccae* NCTC 11659 induces a shift toward a more proactive behavioral coping response during acute exposure to a psychosocial stressor in mice maintained under normal light dark conditions. Future studies are required to determine the mechanisms through which *M. vaccae* NCTC 11659 mediates this stress resilience effect. The effect of *M. vaccae* NCTC 11659 is absent in mice exposed to chronic disruption of rhythms. Nevertheless, immunization with *M. vaccae* NCTC 11659 prevented the effects of circadian misalignment on the alpha diversity of the gut microbiome and brainstem serotonergic systems and therefore could conceivably prevent some negative outcomes of circadian misalignment not measured here, such as colitis (Preuss et al., 2008; Liu et al., 2017; Amara et al., 2019) and affective behavioral responses (Daut et al., 2019).

## Data Availability Statement

The datasets generated for this study can be found in the Qiita (http://qiita.ucsd.edu/study/description/11841), EMBL-EBI (Accession no. ERP015380), and GNPS data repositories (http://massive.ucsd.edu/MSV000082649/ and (http://massive.ucsd.edu/), Dataset Identifiers: MSV000082649 (plasma) and MSV000082650 (fecal). All other data and code may be provided by the corresponding author on reasonable request.

## Ethics Statement

The animal study was reviewed and approved by the University of Colorado Boulder Institutional Animal Care and Use committee and the Bureau of Medicine and Surgery.

## Author Contributions

MV, FT, MF, PD, RK, KPW, and CL designed the research plan and experimental schema. CF, JDH, FV, MB, AE, JS, KML, MA, SS, PS, LM, ML, JEH, DGS, DS, DD, MP, NS, KCW, KN, CG, KS, LT, YM, MO, and JJ conducted the experiments and acquired the data for research analysis. CF, JDH, AG, FV, MB, AE, JS, KML, MA, MCF, SS, LM, ML, JEH, DGS, KAKL, ES, MP, NS, KCW, CS, MV, FT, MF, PD, RK, KPW, and CL analyzed and interpreted the data. CF, JDH, AG, FV, MB, KML, MA, SS, PS, ML, JEH, KAKL, SA, ES, MV, FT, MF, RK, and CL drafted the manuscript. All the authors critically revised and reviewed the manuscript for important intellectual content and agreed to be held accountable to the accuracy and integrity of all work represented here.

## Conflict of Interest

CL serves on the Scientific Advisory Board of Immodulon Therapeutics, Ltd., is cofounder and Chief Scientific Officer of Mycobacteria Therapeutics Corporation, serves as an unpaid scientific consultant with Aurum Switzerland AG and serves at a member of the faculty of the Integrative Psychiatry Institute, Boulder, Colorado, United States. KPW has received research support from the National Institutes of Health, the Pac-12 Conference, and SomaLogic, Inc. outside of this work; consulting fees from or served as a paid member of scientific advisory boards for the Sleep Disorders Research Advisory Board – National Heart, Lung and Blood Institute and CurAegis Technologies, Circadian Therapeutics, Ltd.; and has received speaker/educational/travel consultant honorarium fees from the American Academy of Sleep Medicine, American College of Chest Physicians, American College of Sports Medicine, American Diabetes Association, Associated Professional Sleep Societies, Kellogg Company, and The European Association for the Study of Obesity. The remaining authors declare that the research was conducted in the absence of any commercial or financial relationships that could be construed as a potential conflict of interest.

## Footnotes

1 http://www.earthmicrobiome.org/protocols-and-standards

2 http://qiime2.org

3 http://qiita.ucsd.edu/study/description/11841

4 http://gnps.ucsd.edu

5 http://mzmine.github.io/

6 https://imagej.nih.gov/ij/

7 https://cytoscape.org

## Abbreviations

BBS or Veh: borate-buffered saline vehicle
cDNA: complementary DNA
CDR: chronic disruption of rhythms
CID: collision-induced dissociation
CSC: chronic subordinate colony housing
DDA: data-dependent acquisition
DHA: docosahexaenoic acid
DR: dorsal raphe nucleus
DRC, dorsal raphe nucleus: caudal part
DRD, dorsal raphe nucleus: dorsal part
DRI, dorsal raphe nucleus: interfascicular part
DRV, dorsal raphe nucleus: ventral part
DRVL, dorsal raphe nucleus: ventrolateral part
dsDNA: double-stranded DNA
DSI: Data Sciences International
DSS: dextran sodium sulfate
DTT: dithiothreitol
ESI: electrospray ionization
GNPS: Global Natural Products Social Molecular Networking
GV: gray value
HESI-II: heated electrospray ionization
HMDB: Human Metabolomics Database
IFN γ: interferon gamma
IL-6: interleukin 6
ITI: inter-trial interval
LA: locomotor activity
LC: liquid chromatography
LD: light:dark
LMM: linear mixed model
LSD: least significant difference
LPL-R: lysophospholipid receptor
LPS: lipopolysaccharide
lysoPC: lysophosphatidylcholine
MDT: Mountain Daylight Time
Mfsd2a: major facilitator superfamily domain-containing protein 2A
MnR: median raphe nucleus
mRNA: messenger RNA
MS: mass spectrometry
MST: Mountain Standard Time
*Mv* or *M. vaccae*: *Mycobacterium vaccae* NCTC 11659
NCBI: National Center for Biotechnology Information
NIST: National Institutes of Standards and Technology
NLD: normal light:dark
OLM: object location memory
PCoA: principal coordinates analysis
PCR: polymerase chain reaction
PERMANOVA: permutational analyses of variance
PPAR α: peroxisome proliferator-activated receptor alpha
QIIME2: Quantitative Insights Into Microbial Ecology
R: resolution
REML: restricted maximum likelihood
RF: radio frequency
SD: social defeat
SEM: standard error of the mean
SHC: single-housed home cage control condition
*Slc6a4*: solute carrier family 6 member 4 gene
SSC: saline sodium citrate
*Tph2*,: tryptophan hydroxylase 2 gene
tRNA: transfer ribonucleic acid
Treg: regulatory T cells
UCSD: University of California San Diego
UTC: Coordinated Universal Time
VLPAG: ventrolateral periaqueductal gray
V4: hypervariable region 4
Veh or BBS: borate-buffered saline vehicle
ZT: Zeitgeber time
16S rRNA: 16 small subunit ribosomal RNA.
