# Supplementary material for: Effects of Immunization With the Soil-Derived Bacterium Mycobacterium vaccae on Stress Coping Behaviors and Cognitive Performance in a “Two Hit” Stressor Model
Source: Front Physiol. 2021 Jan 5;11:524833. doi: 10.3389/fphys.2020.524833 (PMC7813891; doi:10.3389/fphys.2020.524833)
Supplement: Supplementary file 2 [file Data_Sheet_1.PDF]

Supplementary Figure 1.

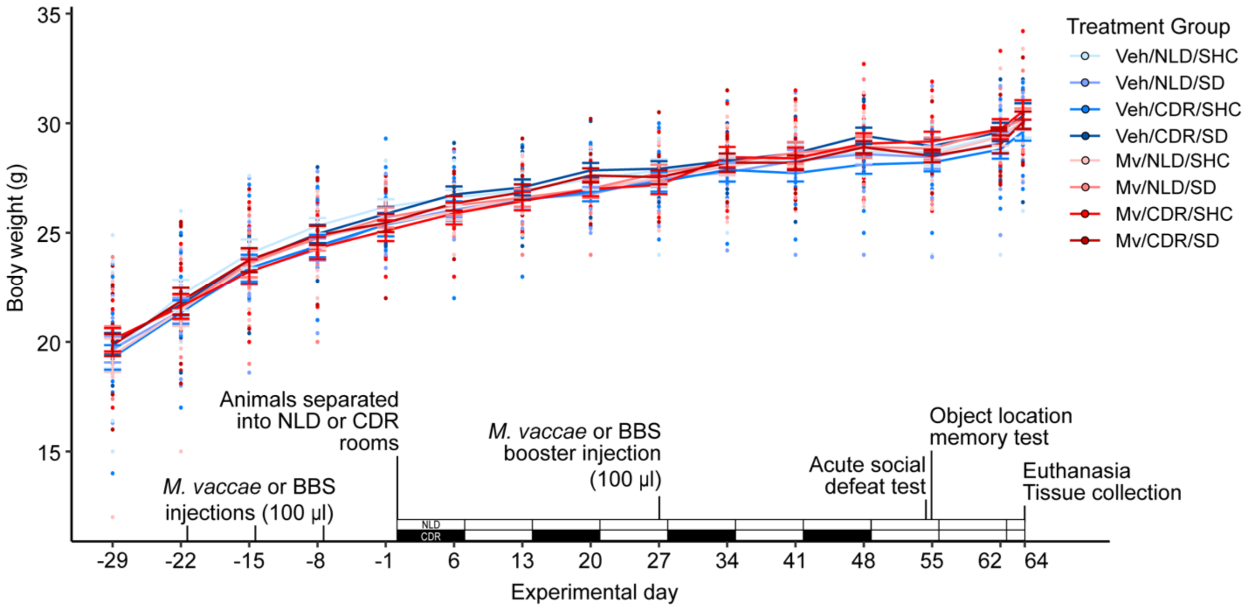

Figure S1. Body weight over time across the experimental protocol. Data represent treatment group means ± standard error of the means (SEM) of mouse body weights (in g), with each data point representing the body weight for an individual mouse at the relevant time point taken during each cage change cycle on days –29, –22, –15, –8, –1, 6, 13, 20, 27, 34, 41, 48, 55, 62, and before tissue collection on day 64. Linear mixed-effects models show no significant differences in body weights due to *Mv* versus Veh treatment, CDR versus NLD, SD versus SHC, or any interactions of the aforementioned factors. White bars indicate normal 12-h light:dark cycling with lights on at 0600 h MDT or 0700 h MST, black bars indicate light cycle reversal periods for the CDR group. Abbreviations: BBS, borate-buffered saline vehicle; CDR, chronic disruption of rhythms; *Mv, Mycobacterium vaccae* NCTC 11659; NLD, normal light:dark condition; SD, social defeat; SHC, single-housed home cage control condition; Veh, borate-buffered saline vehicle.

Supplementary Figure 2

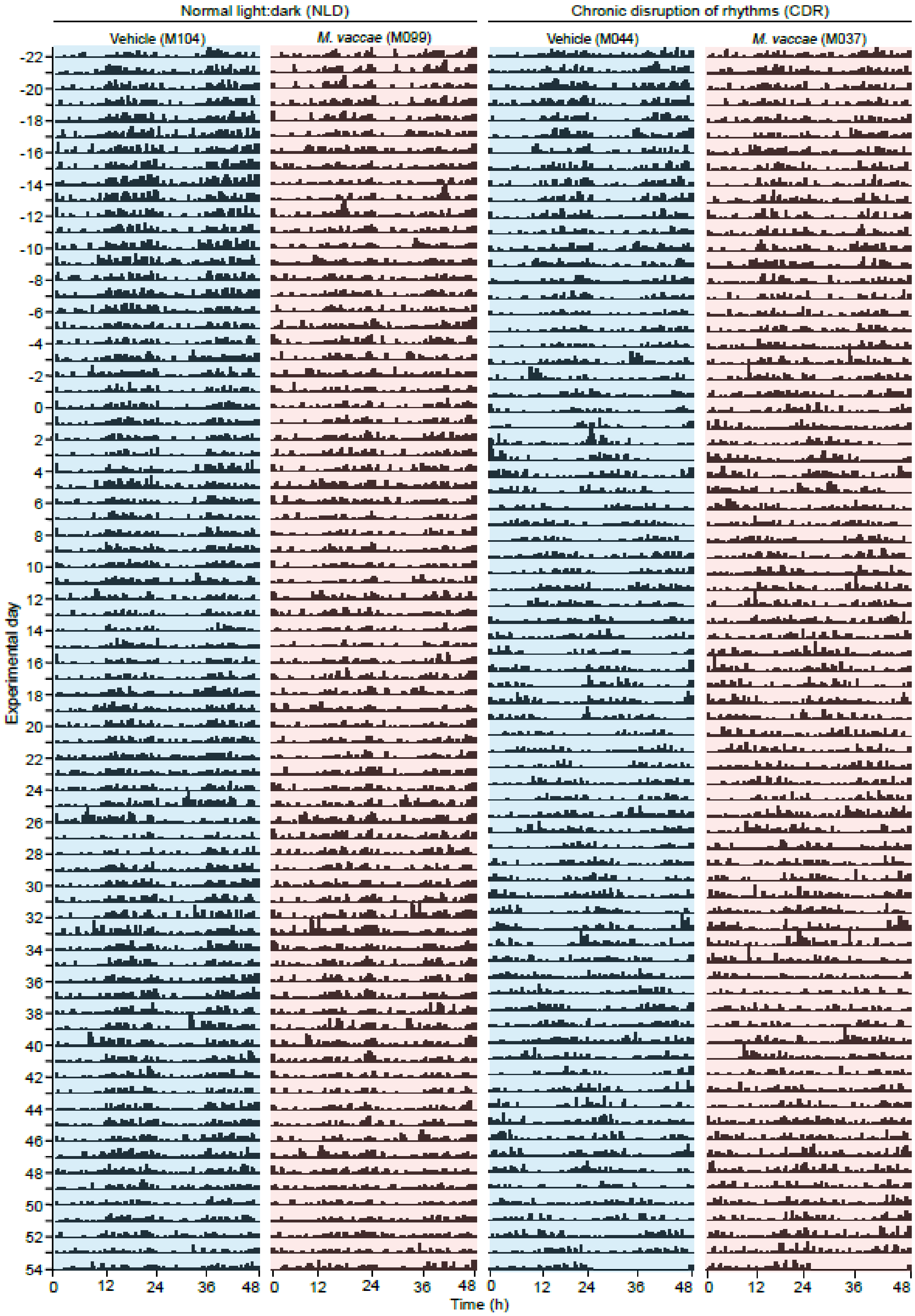

Figure S2. Double-plotted representative actograms illustrating locomotor activity entrainment to daily light cycle cues from day –21 to day 55 for the following groups from left to right: 1) Vehicle group under normal light:dark conditions (NLD); 2) *M. vaccae* group under NLD conditions; 3) Vehicle group under chronic disruption of rhythms (CDR) conditions; and 4) *M. vaccae* group under CDR conditions. Activity is represented as dark areas in each actogram. These data demonstrate that non-shifted mice have stable behavioral patterns while the shifted mice have disrupted behavioral patterns. Increased activity is evident each week before dark onset when experimenters were in the room to collect fecal samples and transfer mice to clean cages. CDR and corresponding NLD control conditions were conducted for the eight-week period from day 0 to day 56. Abbreviations: CDR, chronic disruption of rhythms conditions; *M. vaccae*, *Mycobacterium vaccae* NCTC 11659; NLD, normal light:dark conditions; Vehicle, borate-buffered saline vehicle.

Supplementary Figure 3

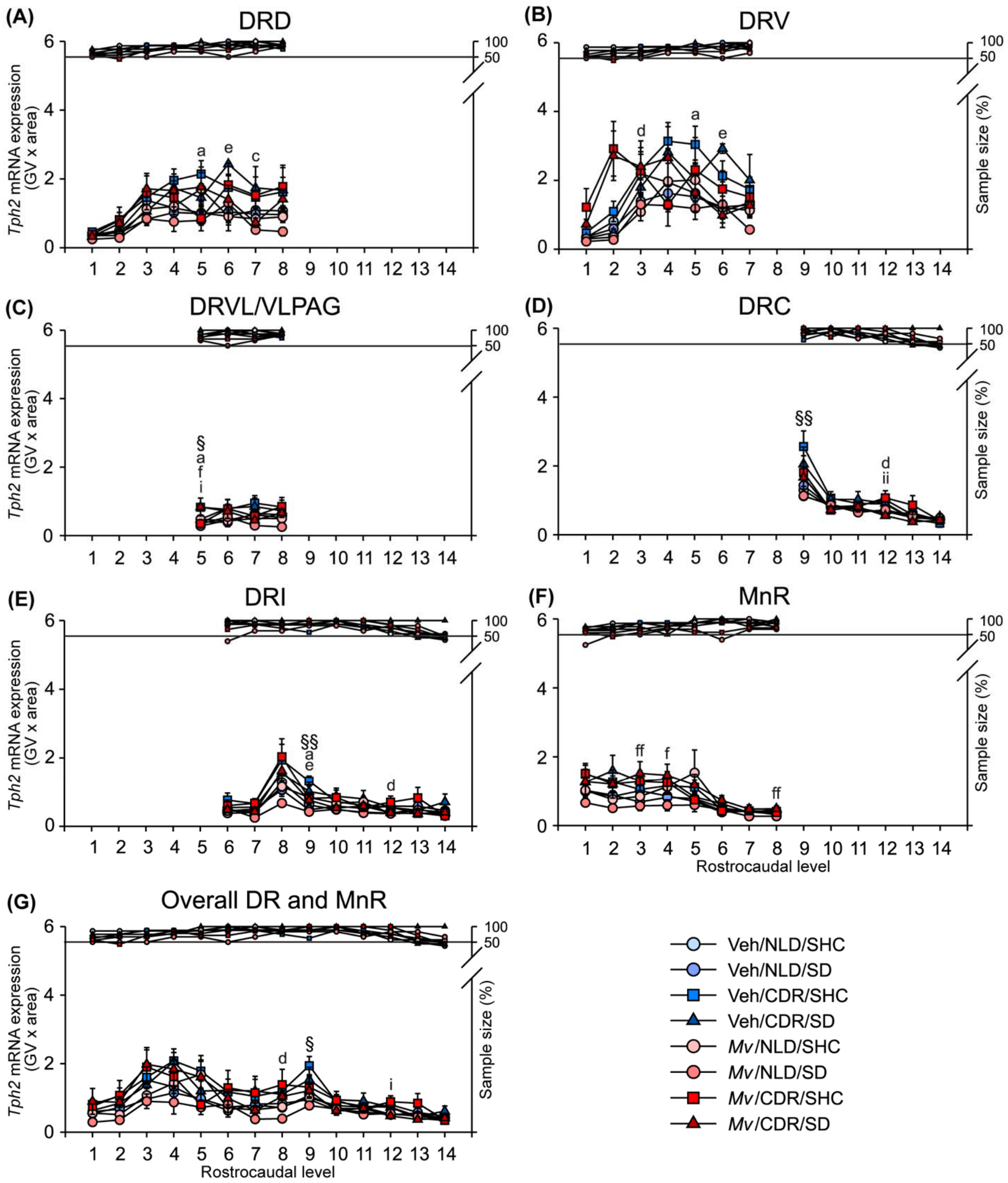

Figure S3. Effects of immunization with *Mycobacterium vaccae* NCTC 11659, chronic disruption of rhythms, and acute social defeat on *Tph2* mRNA expression throughout the rostrocaudal extent of each subregion of the dorsal raphe nucleus (DR) and median raphe nucleus (MnR), as well as the overall DR and MnR combined. Graphs represent the mean ± SEM of

Tph2 mRNA expression in the (A) dorsal raphe nucleus, dorsal part (DRD), (B) dorsal raphe nucleus, ventral part (DRV), (C) dorsal raphe nucleus, ventrolateral part/ventrolateral periaqueductal gray (DRVL/VLPAG), (D) dorsal raphe nucleus, caudal part (DRC), (E) dorsal raphe nucleus, interfascicular part (DRI), (F) MnR, and (G) across all subregions in the DR and MnR. Post hoc comparisons were made using Fisher's least significant difference (LSD) tests; for definitions of symbols used to indicate significant post hoc pairwise comparisons, see Table 4. Post hoc testing was not conducted at a specific rostrocaudal level if one or more groups contained less than 50% of the full sample size at that rostrocaudal level, indicated by the right y-axis. Rostrocaudal levels: 1 = –4.160 mm, 2 = –4.244 mm, 3 = –4.328 mm, 4 = –4.412 mm, 5 = –4.496 mm, 6 = –4.580 mm, 7 = –4.664 mm, 8 = –4.748 mm, 9 = –4.832 mm, 10 = –4.916 mm, 11 = –5.000 mm, 12 = –5.084 mm, 13 = –5.168 mm, 14 = –5.252 mm from bregma. Abbreviations: CDR, chronic disruption of rhythms; DR, dorsal raphe nucleus; DRC, dorsal raphe nucleus, caudal part; DRD, dorsal raphe nucleus, dorsal part; DRI, dorsal raphe nucleus, interfascicular part; DRV, dorsal raphe nucleus, ventral part; DRVL/VLPAG, dorsal raphe nucleus, ventrolateral part/ventrolateral periaqueductal gray; GV, gray value; MnR, median raphe nucleus; Mv, *Mycobacterium vaccae* NCTC 11659; NLD, normal light:dark condition; SD, social defeat; SHC, single-housed home cage control condition; *Tph2*, tryptophan hydroxylase 2; Veh, borate-buffered saline vehicle.

Supplementary Figure 4

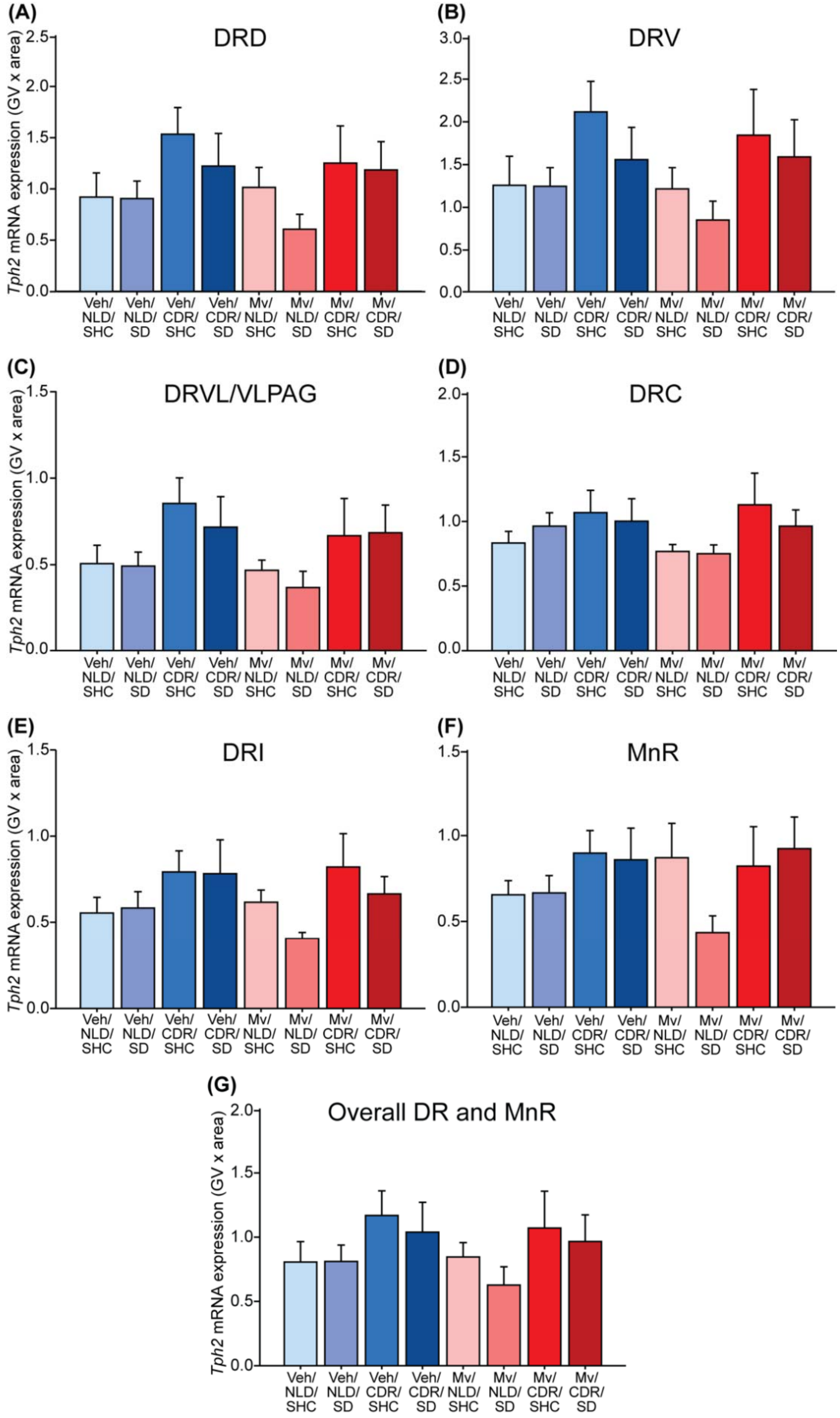

Figure S4. Effects of immunization with *Mycobacterium vaccae* NCTC 11659, chronic disruption of rhythms, and acute social defeat on overall *Tph2* mRNA expression within each subregion of the dorsal raphe nucleus (DR) and median raphe nucleus (MnR), as well as the overall DR and MnR combined. Graphs represent the mean + SEM of *Tph2* mRNA expression in the (A) dorsal raphe nucleus, dorsal part (DRD), (B) dorsal raphe nucleus, ventral part (DRV), (C) dorsal raphe nucleus, ventrolateral part/ventrolateral periaqueductal gray (DRVL/VLPAG), (D) dorsal raphe nucleus, caudal part (DRC), (E) dorsal raphe nucleus, interfascicular part (DRI), (F) MnR, and (G) across all subregions in the DR and MnR. Post hoc comparisons were made using Fisher's least significant difference (LSD) tests; for definitions of symbols used to indicate significant post hoc pairwise comparisons, see Table 4. Abbreviations: CDR, chronic disruption of rhythms; DR, dorsal raphe nucleus; DRC, dorsal raphe nucleus, caudal part; DRD, dorsal raphe nucleus, dorsal part; DRI, dorsal raphe nucleus, interfascicular part; DRV, dorsal raphe nucleus, ventral part; DRVL/VLPAG, dorsal raphe nucleus, ventrolateral part/ventrolateral periaqueductal gray; GV, gray value; MnR, median raphe nucleus; *Mv*, *Mycobacterium vaccae* NCTC 11659; NLD, normal light:dark condition; SD, social defeat; SHC, single-housed home cage control condition; *Tph2*, tryptophan hydroxylase 2; Veh, borate-buffered saline vehicle.

Supplementary Figure 5

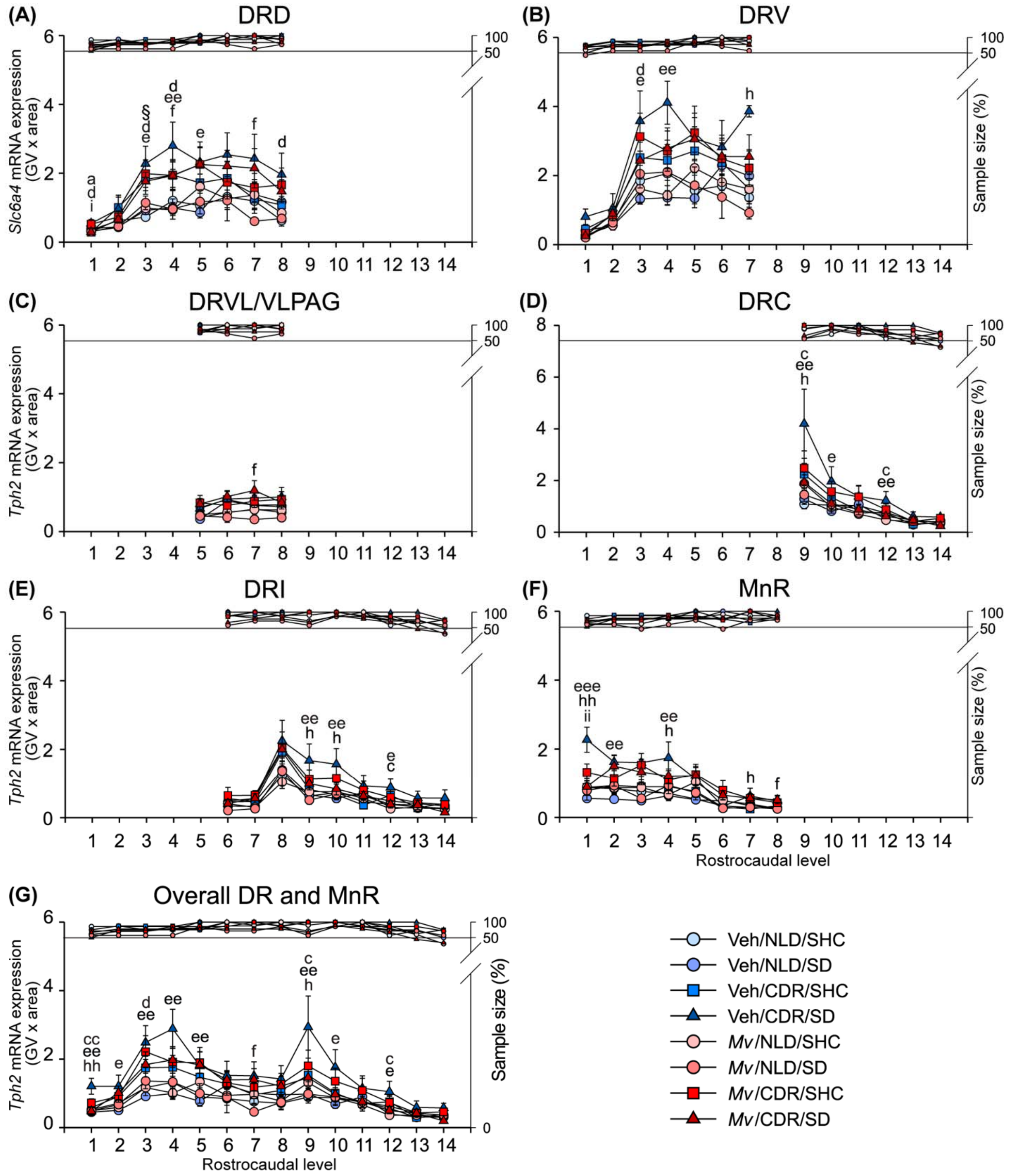

Figure S5. Effects of immunization with *Mycobacterium vaccae* NCTC 11659, chronic disruption of rhythms, and acute social defeat on *Slc6a4* mRNA expression throughout the rostrocaudal extent of each subregion of the dorsal raphe nucleus (DR) and median raphe nucleus (MnR), as well as the overall DR and MnR combined. Graphs represent the mean ± SEM of

Slc6a4 mRNA expression in the (A) dorsal raphe nucleus, dorsal part (DRD), (B) dorsal raphe nucleus, ventral part (DRV), (C) dorsal raphe nucleus, ventrolateral part/ventrolateral periaqueductal gray (DRVL/VLPAG), (D) dorsal raphe nucleus, caudal part (DRC), (E) dorsal raphe nucleus, interfascicular part (DRI), (F) MnR, and (G) across all subregions in the DR and MnR. Post hoc comparisons were made using Fisher's least significant difference (LSD) tests; for definitions of symbols used to indicate significant post hoc pairwise comparisons, see Table 4. Post hoc testing was not conducted at a specific rostrocaudal level if one or more groups contained less than 50% of the full sample size at that rostrocaudal level, indicated by the right y-axis. Rostrocaudal levels: 1 = –4.160 mm, 2 = –4.244 mm, 3 = –4.328 mm, 4 = –4.412 mm, 5 = –4.496 mm, 6 = –4.580 mm, 7 = –4.664 mm, 8 = –4.748 mm, 9 = –4.832 mm, 10 = –4.916 mm, 11 = –5.000 mm, 12 = –5.084 mm, 13 = –5.168 mm, 14 = –5.252 mm from bregma. Abbreviations: CDR, chronic disruption of rhythms; DR, dorsal raphe nucleus; DRC, dorsal raphe nucleus, caudal part; DRD, dorsal raphe nucleus, dorsal part; DRI, dorsal raphe nucleus, interfascicular part; DRV, dorsal raphe nucleus, ventral part; DRVL/VLPAG, dorsal raphe nucleus, ventrolateral part/ventrolateral periaqueductal gray; GV, gray value; MnR, median raphe nucleus; *Mv*, *Mycobacterium vaccae* NCTC 11659; NLD, normal light:dark condition; *Slc6a4*, solute carrier family 6 member 4 (high-affinity, low-capacity sodium-dependent serotonin transporter); SD, social defeat; SHC, single-housed home cage control condition; Veh, borate-buffered saline vehicle.

Supplementary Figure 6

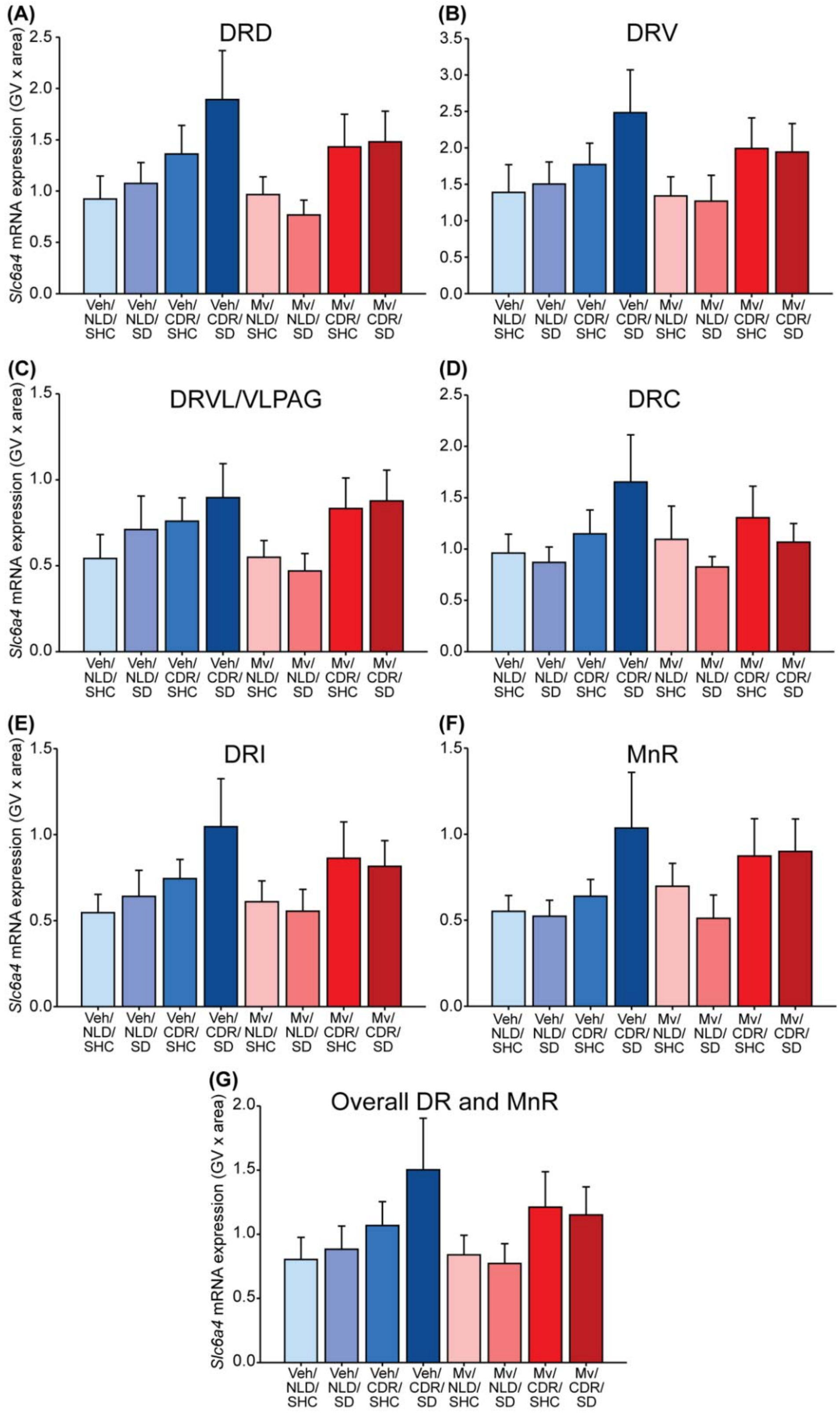

Figure S6. Effects of immunization with *Mycobacterium vaccae* NCTC 11659, chronic disruption of rhythms, and acute social defeat on overall *Slc6a4* mRNA expression within each subregion of the dorsal raphe nucleus (DR) and median raphe nucleus (MnR), as well as the overall DR and MnR combined. Graphs represent the mean ± SEM of *Slc6a4* mRNA expression in the (A) dorsal raphe nucleus, dorsal part (DRD), (B) dorsal raphe nucleus, ventral part (DRV), (C) dorsal raphe nucleus, ventrolateral part/ventrolateral periaqueductal gray (DRVL/VLPAG), (D) dorsal raphe nucleus, caudal part (DRC), (E) dorsal raphe nucleus, interfascicular part (DRI), (F) MnR, and (G) across all subregions in the DR and MnR. Post hoc comparisons were made using Fisher's least significant difference (LSD) tests. Abbreviations: CDR, chronic disruption of rhythms; DR, dorsal raphe nucleus; DRC, dorsal raphe nucleus, caudal part; DRD, dorsal raphe nucleus, dorsal part; DRI, dorsal raphe nucleus, interfascicular part; DRV, dorsal raphe nucleus, ventral part; DRVL/VLPAG, dorsal raphe nucleus, ventrolateral part/ventrolateral periaqueductal gray; GV, gray value; MnR, median raphe nucleus; *Mv*, *Mycobacterium vaccae* NCTC 11659; NLD, normal light:dark condition; *Slc6a4*, solute carrier family 6 member 4 (high-affinity, low-capacity sodium-dependent serotonin transporter); SD, social defeat; SHC, single-housed home cage control condition; Veh, borate-buffered saline vehicle.
