# Supplementary material for: Effects of Immunization With the Soil-Derived Bacterium Mycobacterium vaccae on Stress Coping Behaviors and Cognitive Performance in a “Two Hit” Stressor Model
Source: Front Physiol. 2021 Jan 5;11:524833. doi: 10.3389/fphys.2020.524833 (PMC7813891; doi:10.3389/fphys.2020.524833)
Supplement: Supplementary file 3 [file Data_Sheet_2.PDF]

Supplementary Table S1. Overall linear mixed model analysis for *Tph2* mRNA expression using a first-order ante-dependence covariance structure by REML, selected by best -2 log likelihood criterion.

**Source**	**Test statistic**	***p*-value**
Cohort	$F_{(5, 211.906)} = 31.090$	0.001***
Mycobacterium vaccae NCTC 11659 (*Mv*)	$F_{(1, 258.786)} = 3.665$	0.057
Chronic disruption of rhythms (CDR)	$F_{(1, 309.394)} = 23.620$	0.001***
Acute social defeat (SD)	$F_{(1, 289.818)} = 4.263$	0.040*
Subregion	$F_{(5, 169.079)} = 26.768$	0.001***
Rostrocaudal level	$F_{(13, 200.949)} = 25.673$	0.001***
Mv × Subregion	$F_{(5, 169.077)} = 0.359$	0.876
CDR × Subregion	$F_{(5, 169.077)} = 1.058$	0.385
SD × Subregion	$F_{(5, 169.076)} = 0.274$	0.927
Rostrocaudal level × Subregion	$F_{(23, 221.234)} = 22.749$	0.001***
Mv × CDR	$F_{(1, 260.102)} = 0.019$	0.889
Mv × SD	$F_{(1, 263.026)} = 0.050$	0.823
Mv × Rostrocaudal level	$F_{(13, 200.452)} = 2.014$	0.021*
CDR × SD	$F_{(1, 280.639)} = 0.194$	0.660
CDR × Rostrocaudal level	$F_{(13, 200.561)} = 3.557$	0.001***
SD × Rostrocaudal level	$F_{(13, 200.636)} = 1.452$	0.138
Mv × CDR × Subregion	$F_{(5, 169.074)} = 0.085$	0.995
Mv × SD × Subregion	$F_{(5, 169.076)} = 0.326$	0.897
Mv × CDR × SD	$F_{(1, 271.492)} = 0.093$	0.760
Mv × CDR × Rostrocaudal level	$F_{(13, 200.581)} = 3.474$	0.001***
CDR × SD × Rostrocaudal level	$F_{(13, 200.437)} = 1.462$	0.134
Mv × Subregion × Rostrocaudal level	$F_{(23, 221.225)} = 0.616$	0.916
Mv × CDR × SD × Subregion	$F_{(10, 169.287)} = 0.353$	0.964
Mv × CDR × Subregion × Rostrocaudal level	$F_{(46, 228.603)} = 1.269$	0.132
Mv × CDR × SD × Rostrocaudal level	$F_{(26, 203.560)} = 1.467$	0.075
Mv × CDR × SD × Subregion × Rostrocaudal level	$F_{(92, 226.588)} = 0.386$	1.000
*$p < 0.05$, **$p < 0.01$, *$p < 0.001$		

Supplementary Table S2. Subregional linear mixed model analyses for *Tph2* mRNA expression using unstructured covariance models by REML and best -2 log likelihood criteria.

**Dorsal raphe nucleus, dorsal part**	**Test statistic**	***p*-value**
Cohort	$F_{(5, 19.784)} = 2.121$	0.105
Mycobacterium vaccae NCTC 11659 (*Mv*)	$F_{(1, 42.367)} = 0.54$	0.466
Chronic disruption of rhythms (CDR)	$F_{(1, 43.394)} = 6.191$	0.017*
Acute social defeat (SD)	$F_{(1, 42.635)} = 1.048$	0.312
Rostrocaudal level	$F_{(7, 38.479)} = 12.869$	0.001***
Mv × CDR	$F_{(1, 42.426)} = 0.036$	0.851
Mv × SD	$F_{(1, 42.412)} = 0.001$	0.982
Mv × Rostrocaudal level	$F_{(7, 38.475)} = 1.068$	0.402
CDR × SD	$F_{(1, 42.609)} = 0.025$	0.876

CDR × Rostrocaudal level	$F_{(7, 38.445)} = 1.883$	0.099
SD × Rostrocaudal level	$F_{(7, 38.466)} = 1.090$	0.389
Mv × CDR × SD	$F_{(1, 42.730)} = 0.643$	0.427
CDR × SD × Rostrocaudal level	$F_{(7, 38.460)} = 0.387$	0.904
Mv × CDR × Rostrocaudal level	$F_{(7, 38.492)} = 1.297$	0.278
Mv × CDR × SD × Rostrocaudal level	$F_{(14, 38.396)} = 1.469$	0.170
**Dorsal raphe nucleus, ventral part**		
Cohort	$F_{(5, 23.731)} = 1.684$	0.177
Mycobacterium vaccae NCTC 11659 (*Mv*)	$F_{(1, 42.634)} = 0.594$	0.445
Chronic disruption of rhythms (CDR)	$F_{(1, 43.397)} = 5.7$	0.021*
Acute social defeat (SD)	$F_{(1, 42.844)} = 1.413$	0.241
Rostrocaudal level	$F_{(6, 37.567)} = 18.784$	0.001***
Mv × CDR	$F_{(1, 42.629)} = 0.060$	0.808
Mv × SD	$F_{(1, 42.634)} = 0.013$	0.910
Mv × Rostrocaudal level	$F_{(6, 37.571)} = 1.262$	0.298
CDR × SD	$F_{(1, 42.863)} = 0.017$	0.896
CDR × Rostrocaudal level	$F_{(6, 37.553)} = 1.294$	0.284
SD × Rostrocaudal level	$F_{(6, 37.553)} = 1.431$	0.229
Mv × CDR × SD	$F_{(1, 42.781)} = 0.392$	0.535
CDR × SD × Rostrocaudal level	$F_{(6, 37.570)} = 0.748$	0.615
Mv × CDR × Rostrocaudal level	$F_{(6, 37.557)} = 3.022$	0.016*
Mv × CDR × SD × Rostrocaudal level	$F_{(12, 37.541)} = 1.573$	0.142
**Dorsal raphe nucleus, ventrolateral part/ Ventrolateral periaqueductal grey**		
Cohort	$F_{(5, 43.017)} = 5.736$	0.001***
Mycobacterium vaccae NCTC 11659 (*Mv*)	$F_{(1, 39.746)} = 1.813$	0.186
Chronic disruption of rhythms (CDR)	$F_{(1, 44.11)} = 5.425$	0.024*
Acute social defeat (SD)	$F_{(1, 41.083)} = 0.435$	0.513
Rostrocaudal level	$F_{(3, 44.673)} = 3.650$	0.019*
Mv × CDR	$F_{(1, 39.98)} = 0.131$	0.719
Mv × SD	$F_{(1, 40.431)} = 0.529$	0.471
Mv × Rostrocaudal level	$F_{(3, 44.663)} = 1.855$	0.151
CDR × SD	$F_{(1, 41.118)} = 0.020$	0.889
CDR × Rostrocaudal level	$F_{(3, 44.713)} = 0.340$	0.797
SD × Rostrocaudal level	$F_{(3, 44.662)} = 1.198$	0.321
Mv × CDR × SD	$F_{(1, 41.181)} = 0.188$	0.667
CDR × SD × Rostrocaudal level	$F_{(3, 44.665)} = 0.178$	0.911
Mv × CDR × Rostrocaudal level	$F_{(3, 44.704)} = 0.391$	0.760
Mv × CDR × SD × Rostrocaudal level	$F_{(6, 44.362)} = 1.463$	0.213
**Dorsal raphe nucleus, caudal part**		
Cohort	$F_{(5, 30.659)} = 3.893$	0.008**
Mycobacterium vaccae NCTC 11659 (*Mv*)	$F_{(1, 34.889)} = 2.115$	0.155
Chronic disruption of rhythms (CDR)	$F_{(1, 38.005)} = 6.502$	0.015*
Acute social defeat (SD)	$F_{(1, 35.983)} = 0.224$	0.639

Rostrocaudal level	$F_{(5, 27.88)} = 52.541$	0.001***
Mv × CDR	$F_{(1, 34.554)} = 0.328$	0.571
Mv × SD	$F_{(1, 35.305)} = 0.008$	0.931
Mv × Rostrocaudal level	$F_{(5, 27.868)} = 2.671$	0.043*
CDR × SD	$F_{(1, 36.132)} = 1.473$	0.233
CDR × Rostrocaudal level	$F_{(5, 27.887)} = 3.816$	0.009**
SD × Rostrocaudal level	$F_{(5, 27.895)} = 0.506$	0.769
Mv × CDR × SD	$F_{(1, 36.077)} = 0.730$	0.399
CDR × SD × Rostrocaudal level	$F_{(5, 27.903)} = 2.069$	0.100
Mv × CDR × Rostrocaudal level	$F_{(5, 27.878)} = 3.279$	0.019*
Mv × CDR × SD × Rostrocaudal level	$F_{(10, 27.900)} = 0.820$	0.613
**Dorsal raphe nucleus, interfascicular part**		
Cohort	$F_{(5, 37.701)} = 11.198$	0.001***
Mycobacterium vaccae NCTC 11659 (*Mv*)	$F_{(1, 37.738)} = 1.038$	0.315
Chronic disruption of rhythms (CDR)	$F_{(1, 44.437)} = 9.975$	0.003**
Acute social defeat (SD)	$F_{(1, 38.795)} = 1.323$	0.257
Rostrocaudal level	$F_{(8, 32.831)} = 12.876$	0.001***
Mv × CDR	$F_{(1, 37.880)} = 0.167$	0.685
Mv × SD	$F_{(1, 38.778)} = 0.628$	0.433
Mv × Rostrocaudal level	$F_{(8, 32.822)} = 0.533$	0.823
CDR × SD	$F_{(1, 38.836)} = 0.068$	0.795
CDR × Rostrocaudal level	$F_{(8, 32.751)} = 3.110$	0.010*
SD × Rostrocaudal level	$F_{(8, 32.678)} = 1.048$	0.422
Mv × CDR × SD	$F_{(1, 39.377)} = 0.115$	0.736
CDR × SD × Rostrocaudal level	$F_{(8, 32.742)} = 1.535$	0.183
Mv × CDR × Rostrocaudal level	$F_{(8, 32.837)} = 2.782$	0.018*
Mv × CDR × SD × Rostrocaudal level	$F_{(16, 32.310)} = 0.944$	0.533
**Median raphe nucleus**		
Cohort	$F_{(5, 39.975)} = 3.024$	0.021*
Mycobacterium vaccae NCTC 11659 (*Mv*)	$F_{(1, 24.542)} = 0.114$	0.739
Chronic disruption of rhythms (CDR)	$F_{(1, 25.475)} = 1.736$	0.199
Acute social defeat (SD)	$F_{(1, 24.829)} = 0.836$	0.369
Rostrocaudal level	$F_{(7, 31.870)} = 11.334$	0.001***
Mv × CDR	$F_{(1, 24.677)} = 0.058$	0.811
Mv × SD	$F_{(1, 24.705)} = 0.798$	0.380
Mv × Rostrocaudal level	$F_{(7, 31.974)} = 1.613$	0.168
CDR × SD	$F_{(1, 24.848)} = 1.731$	0.200
CDR × Rostrocaudal level	$F_{(7, 31.946)} = 0.947$	0.485
SD × Rostrocaudal level	$F_{(7, 31.991)} = 1.204$	0.329
Mv × CDR × SD	$F_{(1, 24.900)} = 1.035$	0.319
CDR × SD × Rostrocaudal level	$F_{(7, 31.969)} = 1.672$	0.152
Mv × CDR × Rostrocaudal level	$F_{(7, 32.003)} = 0.973$	0.467
Mv × CDR × SD × Rostrocaudal level	$F_{(14, 31.232)} = 0.458$	0.939

*p < 0.05, **p < 0.01, *p < 0.001

Supplementary Table S3. Overall linear mixed model analysis for *Slc6a4* mRNA expression using a first-order ante-dependence covariance structure by REML, selected by best -2 log likelihood criterion.

**Source**	**Test statistic**	***p*-value**
Cohort	$F_{(5, 119.812)} = 20.467$	.000***
Mycobacterium vaccae NCTC 11659 (*Mv*)	$F_{(1, 286.887)} = 2.513$	.114
Chronic disruption of rhythms (CDR)	$F_{(1, 356.592)} = 45.539$	.000***
Acute social defeat (SD)	$F_{(1, 327.888)} = 1.984$	.160
Subregion	$F_{(5, 156.763)} = 32.663$	.001***
Rostrocaudal level	$F_{(13, 213.074)} = 46.419$	.001***
Mv × Subregion	$F_{(5, 156.766)} = 0.322$	.899
CDR × Subregion	$F_{(5, 156.764)} = 1.461$	.206
SD × Subregion	$F_{(5, 156.763)} = 0.198$	.963
Rostrocaudal level × Subregion	$F_{(23, 268.966)} = 30.625$	.001***
Mv × CDR	$F_{(1, 289.011)} = 0.290$	.591
Mv × SD	$F_{(1, 286.801)} = 5.369$	.021*
Mv × Rostrocaudal level	$F_{(13, 212.975)} = 2.256$	.009**
CDR × SD	$F_{(1, 313.071)} = 4.867$	.028*
CDR × Rostrocaudal level	$F_{(13, 212.922)} = 4.854$	.001***
SD × Rostrocaudal level	$F_{(13, 212.603)} = 1.398$	.162
Mv × CDR × Subregion	$F_{(5, 156.766)} = 0.104$	.991
Mv × SD × Subregion	$F_{(5, 156.765)} = 0.291$	.917
Mv × CDR × SD	$F_{(1, 299.027)} = 7.710$	.006**
Mv × CDR × Rostrocaudal level	$F_{(13, 212.832)} = 1.490$	.123
CDR × SD × Rostrocaudal level	$F_{(13, 212.683)} = 1.902$	.031*
Mv × Subregion × Rostrocaudal level	$F_{(23, 268.963)} = 0.590$	.934
Mv × CDR × SD × Subregion	$F_{(10, 157.013)} = 0.195$	.996
Mv × CDR × Subregion × Rostrocaudal level	$F_{(46, 268.202)} = 1.345$	.079
Mv × CDR × SD × Rostrocaudal level	$F_{(26, 212.473)} = 2.061$	.003**
Mv × CDR × SD × Subregion × Rostrocaudal level	$F_{(92, 265.55)} = 0.721$	.966
*p < 0.05, **p < 0.01, *p < 0.001		

Supplementary Table S4. Subregional linear mixed model analyses for *Slc6a4* mRNA expression using unstructured covariance models by REML and best -2 log likelihood criteria.

**Dorsal raphe nucleus, dorsal part**	**Test statistic**	***p*-value**
Cohort	$F_{(5, 27.194)} = 2.477$	0.057
Mycobacterium vaccae NCTC 11659 (*Mv*)	$F_{(1, 43.808)} = 0.526$	0.472
Chronic disruption of rhythms (CDR)	$F_{(1, 45.978)} = 13.306$	0.001***
Acute social defeat (SD)	$F_{(1, 44.112)} = 0.421$	0.520
Rostrocaudal level	$F_{(7, 40.513)} = 33.702$	0.001***
Mv × CDR	$F_{(1, 43.878)} = 0.001$	0.981
Mv × SD	$F_{(1, 43.862)} = 0.923$	0.342

Mv × Rostrocaudal level	$F_{(7, 40.363)} = 1.992$	0.080
CDR × SD	$F_{(1, 44.036)} = 0.917$	0.344
CDR × Rostrocaudal level	$F_{(7, 40.459)} = 5.477$	0.001***
SD × Rostrocaudal level	$F_{(7, 40.340)} = 1.219$	0.315
Mv × CDR × SD	$F_{(1, 44.489)} = 0.128$	0.722
CDR × SD × Rostrocaudal level	$F_{(7, 40.332)} = 0.525$	0.810
Mv × CDR × Rostrocaudal level	$F_{(7, 40.408)} = 0.321$	0.940
Mv × CDR × SD × Rostrocaudal level	$F_{(14, 40.529)} = 0.898$	0.567
**Dorsal raphe nucleus, ventral part**		
Cohort	$F_{(5, 28.039)} = 2.711$	0.040*
Mycobacterium vaccae NCTC 11659 (*Mv*)	$F_{(1, 45.882)} = 0.51$	0.479
Chronic disruption of rhythms (CDR)	$F_{(1, 46.823)} = 9.29$	0.004**
Acute social defeat (SD)	$F_{(1, 46.054)} = 0.533$	0.469
Rostrocaudal level	$F_{(6, 40.270)} = 34.237$	0.001***
Mv × CDR	$F_{(1, 45.924)} = 0.004$	0.951
Mv × SD	$F_{(1, 45.897)} = 0.851$	0.361
Mv × Rostrocaudal level	$F_{(6, 40.253)} = 0.884$	0.515
CDR × SD	$F_{(1, 45.988)} = 0.376$	0.543
CDR × Rostrocaudal level	$F_{(6, 40.253)} = 3.378$	0.009**
SD × Rostrocaudal level	$F_{(6, 40.227)} = 1.552$	0.186
Mv × CDR × SD	$F_{(1, 46.171)} = 0.294$	0.590
CDR × SD × Rostrocaudal level	$F_{(6, 40.252)} = 0.856$	0.535
Mv × CDR × Rostrocaudal level	$F_{(6, 40.230)} = 0.573$	0.749
Mv × CDR × SD × Rostrocaudal level	$F_{(12, 40.256)} = 1.322$	0.244
**Dorsal raphe nucleus, ventrolateral part/ Ventrolateral periaqueductal grey**		
Cohort	$F_{(5, 42.157)} = 13.173$	0.001***
Mycobacterium vaccae NCTC 11659 (*Mv*)	$F_{(1, 46.626)} = 0.396$	0.532
Chronic disruption of rhythms (CDR)	$F_{(1, 51.324)} = 3.998$	0.051
Acute social defeat (SD)	$F_{(1, 47.330)} = 0.182$	0.672
Rostrocaudal level	$F_{(3, 47.492)} = 5.661$	0.002**
Mv × CDR	$F_{(1, 46.775)} = 1.440$	0.236
Mv × SD	$F_{(1, 47.047)} = 0.184$	0.670
Mv × Rostrocaudal level	$F_{(3, 47.475)} = 0.808$	0.496
CDR × SD	$F_{(1, 47.286)} = 0.137$	0.713
CDR × Rostrocaudal level	$F_{(3, 47.357)} = 0.160$	0.923
SD × Rostrocaudal level	$F_{(3, 47.399)} = 0.368$	0.776
Mv × CDR × SD	$F_{(1, 48.036)} = 0.052$	0.821
CDR × SD × Rostrocaudal level	$F_{(3, 47.403)} = 0.790$	0.505
Mv × CDR × Rostrocaudal level	$F_{(3, 47.429)} = 0.799$	0.501
Mv × CDR × SD × Rostrocaudal level	$F_{(6, 47.529)} = 0.708$	0.645
**Dorsal raphe nucleus, caudal part**		
Cohort	$F_{(5, 24.962)} = 10.162$	0.001***
Mycobacterium vaccae NCTC 11659 (*Mv*)	$F_{(1, 43.422)} = 0.919$	0.343

Chronic disruption of rhythms (CDR)	$F_{(1, 45.568)} = 7.038$	0.011*
Acute social defeat (SD)	$F_{(1, 43.419)} = 0.168$	0.684
Rostrocaudal level	$F_{(5, 39.587)} = 20.033$	0.001***
Mv × CDR	$F_{(1, 43.402)} = 1.896$	0.176
Mv × SD	$F_{(1, 43.495)} = 2.432$	0.126
Mv × Rostrocaudal level	$F_{(5, 39.756)} = 1.693$	0.159
CDR × SD	$F_{(1, 43.437)} = 0.590$	0.447
CDR × Rostrocaudal level	$F_{(5, 39.705)} = 2.223$	0.071
SD × Rostrocaudal level	$F_{(5, 39.468)} = 0.602$	0.699
Mv × CDR × SD	$F_{(1, 43.669)} = 5.705$	0.021*
CDR × SD × Rostrocaudal level	$F_{(5, 39.520)} = 1.300$	0.283
Mv × CDR × Rostrocaudal level	$F_{(5, 39.722)} = 2.117$	0.083
Mv × CDR × SD × Rostrocaudal level	$F_{(10, 39.399)} = 1.881$	0.078
**Dorsal raphe nucleus, interfascicular part**		
Cohort	$F_{(5, 39.994)} = 4.533$	0.002**
Mycobacterium vaccae NCTC 11659 (*Mv*)	$F_{(1, 34.006)} = 0.309$	0.582
Chronic disruption of rhythms (CDR)	$F_{(1, 37.093)} = 7.705$	0.009**
Acute social defeat (SD)	$F_{(1, 34.167)} = 0.447$	0.508
Rostrocaudal level	$F_{(8, 37.499)} = 15.442$	0.001***
Mv × CDR	$F_{(1, 34.038)} = 0.236$	0.630
Mv × SD	$F_{(1, 34.092)} = 1.75$	0.195
Mv × Rostrocaudal level	$F_{(8, 37.563)} = 1.794$	0.109
CDR × SD	$F_{(1, 34.250)} = 0.241$	0.627
CDR × Rostrocaudal level	$F_{(8, 37.475)} = 3.038$	0.010*
SD × Rostrocaudal level	$F_{(8, 37.430)} = 2.126$	0.058
Mv × CDR × SD	$F_{(1, 34.551)} = 1.663$	0.206
CDR × SD × Rostrocaudal level	$F_{(8, 37.461)} = 3.26$	0.006**
Mv × CDR × Rostrocaudal level	$F_{(8, 37.608)} = 1.237$	0.305
Mv × CDR × SD × Rostrocaudal level	$F_{(16, 37.432)} = 2.954$	0.003**
**Median raphe nucleus**		
Cohort	$F_{(5, 30.960)} = 1.625$	0.183
Mycobacterium vaccae NCTC 11659 (*Mv*)	$F_{(1, 38.743)} = 0.223$	0.640
Chronic disruption of rhythms (CDR)	$F_{(1, 41.042)} = 9.004$	0.005**
Acute social defeat (SD)	$F_{(1, 39.010)} = 0.617$	0.437
Rostrocaudal level	$F_{(7, 35.622)} = 22.651$	0.001***
Mv × CDR	$F_{(1, 38.833)} = 0.027$	0.871
Mv × SD	$F_{(1, 38.683)} = 2.059$	0.159
Mv × Rostrocaudal level	$F_{(7, 35.561)} = 3.03$	0.013*
CDR × SD	$F_{(1, 39.016)} = 2.468$	0.124
CDR × Rostrocaudal level	$F_{(7, 35.546)} = 1.628$	0.159
SD × Rostrocaudal level	$F_{(7, 35.460)} = 1.898$	0.099
Mv × CDR × SD	$F_{(1, 39.320)} = 0.872$	0.356
CDR × SD × Rostrocaudal level	$F_{(7, 35.507)} = 0.751$	0.631

Mv × CDR × Rostrocaudal level	$F_{(7, 35.531)} = 1.372$	0.247
Mv × CDR × SD × Rostrocaudal level	$F_{(14, 35.378)} = 1.888$	0.063
*$p < 0.05$, **$p < 0.01$, *$p < 0.001$		

Supplementary File S1. Data inclusion criteria table listing data used for analysis throughout 2015–2017 and calculated *n* values for each analysis.
